# Pain can’t be carved at the joints: defining function-based pain profiles and their relevance to chronic disease management in healthcare delivery design

**DOI:** 10.1186/s12916-024-03807-z

**Published:** 2024-12-18

**Authors:** Daniel S. Barron, Karin Saltoun, Hannah Kiesow, Melanie Fu, Jessica Cohen-Tanugi, Paul Geha, Dustin Scheinost, Zacharia Isaac, David Silbersweig, Danilo Bzdok

**Affiliations:** 1 Department of Psychiatry, Brigham & Women’s Hospital, Mass General Brigham, Boston, USA; 2 Department of Physical Medicine and Rehabilitation, Spaulding Rehabilitation Hospital, Mass General Brigham, Boston, USA; 3 Department of Biomedical Engineering, Montreal Neurological Institute, McGill University and Mila – Quebec AI Institute, Montreal, Canada; 4 Harvard University, Boston, USA; 5 Departments of Neuroscience, Psychiatry, Dentistry and Neurology, University of Rochester, Rochester, USA; 6 Department of Radiology, Yale University, New Haven, USA

**Keywords:** Pain, Chronic pain, Chronic disease management, Big data, Healthcare delivery design, Machine learning, UK Biobank

## Abstract

**Background:**

Pain is a complex problem that is triaged, diagnosed, treated, and billed based on which body part is painful, almost without exception. While the “body part framework” guides the organization and treatment of individual patients’ pain conditions, it remains unclear how to best conceptualize, study, and treat pain conditions at the population level. Here, we investigate (1) how the body part framework agrees with population-level, biologically derived pain profiles; (2) how do data-derived pain profiles interface with other symptom domains from a whole-body perspective; and (3) whether biologically derived pain profiles capture clinically salient differences in medical history.

**Methods:**

To understand how pain conditions might be best organized, we applied a carefully designed a multi-variate pattern-learning approach to a subset of the UK Biobank (*n* = 34,337), the largest publicly available set of real-world pain experience data to define common population-level profiles. We performed a series of post hoc analyses to validate that each pain profile reflects real-world, clinically relevant differences in patient function by probing associations of each profile across 137 medication categories, 1425 clinician-assigned ICD codes, and 757 expert-curated phenotypes.

**Results:**

We report four unique, biologically based pain profiles that cut across medical specialties: pain interference, depression, medical pain, and anxiety, each representing different facets of functional impairment. Importantly, these profiles do not specifically align with variables believed to be important to the standard pain evaluation, namely painful body part, pain intensity, sex, or BMI. Correlations with individual-level clinical histories reveal that our pain profiles are largely associated with clinical variables and treatments of modifiable, chronic diseases, rather than with specific body parts. Across profiles, notable differences include opioids being associated only with the pain interference profile, while antidepressants linked to the three complimentary profiles. We further provide evidence that our pain profiles offer valuable, additional insights into patients’ wellbeing that are not captured by the body-part framework and make recommendations for how our pain profiles might sculpt the future design of healthcare delivery systems.

**Conclusion:**

Overall, we provide evidence for a shift in pain medicine delivery systems from the conventional, body-part-based approach to one anchored in the pain experience and holistic profiles of patient function. This transition facilitates a more comprehensive management of chronic diseases, wherein pain treatment is integrated into broader health strategies. By focusing on holistic patient profiles, our approach not only addresses pain symptoms but also supports the management of underlying chronic conditions, thereby enhancing patient outcomes and improving quality of life. This model advocates for a seamless integration of pain management within the continuum of care for chronic diseases, emphasizing the importance of understanding and treating the interdependencies between chronic conditions and pain.

**Supplementary Information:**

The online version contains supplementary material available at 10.1186/s12916-024-03807-z.

## Background

**Fig. 1:**
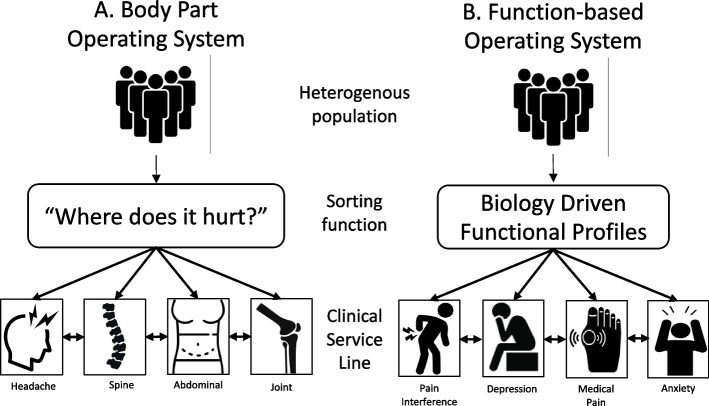
Operating systems in healthcare delivery: body-part and function-based frameworks. We use the term “operating system” to describe the “sequence of decisions and tasks needed to address a health problem” ([1], pg 58). Practically, operating system describes how a patient enters, is sorted by, and arrives at a clinical service line where they receive care for their specific health problem. The sorting function plays a key role in framing the patient’s health problem and in organizing the specialized clinical workforce to address that health problem. Pain medicine operates within a body-part framework (**A**): when a patient enters the healthcare delivery system, they are asked which body part is painful. Based on the patient’s answer, the healthcare system sorts the patient to the clinical service line that focuses on that body part (e.g., headache clinic versus a spine service line). The painful body part, therefore, is a sorting function that determines not only the clinical service line, but also the skillset of the clinical workforce where the patient is evaluated and treated. Data from the UK Biobank suggests that the body part operating system does not fully capture health problems or pain experience across 34,337 participants. We report biologically grounded pain profiles that could sort connect patients with clinical service lines organized around functional status (**B**). Each pain profile broadly tracks a different domain of function: pain interference, depression, medical pain, and anxiety. Function-based profiles could see beyond specific body parts and sort patients into a care delivery system that address patients’ overall health. (Adapted from [1], Fig. 5.3. *We do not have permission to use these clipart (which we found by google images) but present them as conceptual place holders; we are happy to work with the journal’s graphic designer of choice to improve this figure.)*

“Where does it hurt?”—the most common starting point for the clinical exploration of pain. Patients are triaged, diagnosed, treated, and even billed, all according to which body part is painful (see Fig. 1). Body parts function as an operational tool at the heart of modern healthcare delivery systems: patients are evaluated and ushered towards the clinical service lines best equipped to address health problems of that specific body part [1]. Within this body-part framework, clinical specialists apply existing knowledge to treat a body part and researchers study how to detect, stage, or improve damage to that body part. Yet, when it comes to an individual’s pain, the question “where does it hurt?” can be misleading.

Pain is a complex, pervasive issue affecting approximately 25% of adults and incurs an annual economic burden of around $560 billion in the USA [2, 3]. While not directly fatal, pain contributes to diminished physical function, psychiatric conditions such as depression and anxiety, and risks associated with pain medication, including addiction [2]. Paradoxically, pain can exist with minimal detectable physical damage, and significant damage can occur without pain. For instance, up to 96% of people show MRI evidence of spine degeneration without experiencing back pain, and rotator cuff tears often coexist with normal, painless function [4–7]. This disconnect highlights that pain perception extends beyond observable physical injury, involving complex biological, psychological, and social factors [8].

Although clinically, the reflex is to look for tissue damage as a cause of pain, the genetic, inflammatory, and neurophysiologic mechanisms that modify pain can be unrelated to tissue damage [8, 9]. Psychological factors such as depression and anxiety impact pain perception and chronicity; pain is so commonly observed with depression and anxiety, they could be part of the same disease process [10, 11]. Among 5381 pain patients with low back pain, 32% were diagnosed with either depression or anxiety and 47% had both depression and anxiety [12]. Notably, while patients with chronic low back pain respond to so-called “analgesic” anti-depressants (e.g., serotonin-norepinephrine reuptake inhibitors), treatments that might help acute pain in the same body part (e.g., opioids) are not recommended. Social determinants, including socioeconomic status and social support, influence pain development and outcomes [13–15]. Lower socioeconomic status, social isolation, and poorer support networks are linked to higher levels of pain and disability, and the reverse is true for improved pain. While biologic roots no doubt can mediate these factors, the incarnation of social determinants is most likely not in a specific body part, but instead diffused throughout the body’s nervous, immune, and cardiovascular systems in ways that likely differ for different conditions in different people.

Given the vast heterogeneity of pain’s origins, presentation, treatments, and causes (and we have but scratched the surface here), it is no wonder that decades of pain research hinging on a body-part logic have not revealed how to best conceptualize, study, and treat pain conditions at the population level. It is reasonable to consider that attempts to better understand pain conditions might embrace the complexity of how pain conditions present, are described, and treated across a wide population. From a clinical perspective, in other words, if medical care is the organized delivery of science [1], how can pain medicine best be organized?

Addressing how pain medicine can be organized more effectively at the population level could represent a crucial first step in standardizing and improving care. That is, in addition to *describing* the population-level features of pain, such an organization might suggest modifiable practices for the treatment of pain conditions, including how pain conditions are reimbursed. Currently, most payors reimburse costly procedures based on a body-part framework, such as lumbar epidural steroid injections for back pain. However, as recently noted by the American Academy of Pain Medicine [16], these same payors often do not reimburse (or reimburse poorly) clinical services that address associated conditions like depression or anxiety. This discrepancy underscores the need for a more integrated approach to pain management that considers the full spectrum of factors influencing pain.

Multivariate methods are increasingly deployed to understand biologic variability in the context of pain experience and overall functional status. In migraine research, for example, a multimodal factor mixture model was used to classify patients based on structural MRIs, revealing migraine subtypes with distinct pain experience, and level of disability [17]. A separate study used partial least squares correlation and hierarchical analysis to classify patients based on MRI-based functional connectivity into subgroups with distinct depression severity and treatment response to electroacupuncture [18]. In pain following spine surgery, multivariate tools have been applied to identify perioperative factors that predict acute pain level, demonstrating the importance of using multiple nonopioid analgesic classes during surgery to reduce postoperative pain [19]. A compelling 2023 study published in Nature Medicine applied multivariate machine learning algorithms to 99 pain-agnostic, physical, psychological, demographic, and social factors from the UK Biobank to define a prognostic risk score that classified various chronic pain conditions and predicted the development of widespread chronic pain across different body sites [20]. These studies collectively demonstrate the significant advances that multivariate approaches have brought to pain medicine, suggesting that more personalized and effective treatment options can be born of comprehensive data analysis.

Here, we apply multivariate methods to the UK Biobank—the world’s largest biomedical dataset with up to 500,000 participants—to investigate pain’s complexity in a holistic fashion: using a variety of measures, including real-time functional assessments, high-quality brain imaging scans, and clinical histories. We deploy multivariate machine learning methods to the UK Biobank’s population-scale profiling to first, define the most common patterns of pain experience across the population. So defined, we then ask 3 questions: (1) do individual pain profiles capture clinically relevant differences in pain symptomatology? (2) do biologically derived pain profiles capture clinically salient differences in medical history? (3) how does the dominant focus on a painful body part compare with population-level, biologically derived pain profiles? We report that, across the wider UK population, four dominant pain profiles emerge that might offer new organizing principles for the evaluation and treatment of pain conditions, of relevance to chronic disease management across all specialties of medicine and to the on-going and future design of healthcare delivery systems.

## Methods

### Human population data source

The UK Biobank is a prospective epidemiological resource that provides a rich palette of information including high-quality brain imaging, genetics, and various biological and lifestyle measurements in a cohort of ∼500,000 participants recruited from across the UK, following ~ 9.2 million invitations sent to individuals registered with the UK’s National Health Service who were aged 40–69 years and lived within approximately 25 miles (40 km) of one of 22 assessment centers located throughout England, Wales, and Scotland [21]. Further information on the consent procedure can be referenced at biobank.ctsu.ox.ac.uk/crystal/field.cgi?id = 200. Analysis of UK Biobank non-identifiable data received Institutional Review Board (IRB) approval from MassGeneralBrigham and McGill IRBs as Not Human Subjects Research. Participants were aged 40–69 years when recruited (mean age 55, standard deviation 7.5 years). We focused on high-resolution T1-weighted structural brain scans the February 2020 data release of 40,681 (54% female) participants (see Supplementary Table 10 regarding demographic information of final sample, described below). UK Biobank data are available through a procedure described at http://www.ukbiobank.ac.uk/using-the-resource/. The present analyses were conducted under UK Biobank application number 25163, “Structural genetic contributions brain function and behavior.” Before proceeding to mine the UKB for genetic contributions to pain condition (which, as stated above, have a high overlap with depression and anxiety), the present work is a first step towards more clearly defining population-level patterns of pain behavior that are anchored in biology. So defined, we expect that our pain profiles will facilitate later studies of the genetic architecture of pain conditions and, as focused on in this manuscript, guide clinical operations.

For the benefit of reproducibility and comparability, all our analyses of brain–behavior association were based on the precomputed and expert-vetted image-derived phenotypes [22]. For our analyses of gray matter structure, we relied on volume estimates in 100 cortical regions defined by the Schaefer-Yeo atlas (see Supplementary Table 5) [23]. All structural magnetic resonance imaging (MRI) data were preprocessed using the pipelines and quality-control workflows by the FMRIB team, Oxford University, UK [24]. The uniform preprocessing increases the comparability of our findings to other UK Biobank studies. We used common linear deconfounding to remove variation in all brain-imaging-derived phenotypes that could be explained by interindividual differences in head size and motion, following previously published UK Biobank research [25]. We applied the same normalization procedures to the pain experience variables, described below. As such, effects emerging from the subsequent modeling steps on the thus cleaned brain phenotypes cannot be explained by differences in brain size or motion.

Our primary goal was to define clinically relevant population-level modes of variability that as associated with pain conditions. As it is known that age [26], body mass index [27], and sex [28, 29] are associated with clinical pain in biologically and clinically important ways, we decided not to deconfound these variables from brain imaging-derived target measures with the logic that deconfounding would remove precisely the variability we were hoping to exploit to define clinically relevant population-level modes of co-variation. We further note that, consistent with our previous work, we have not regressed out sociodemographic variables with the goal of preserving rich insights into brain and behavior, by interlocking effects with other variables of interest [30–33].

Of the 40,681 UK Biobank participants with brain imaging data, 38,692 participants also had pain experience data (see Supplementary Table 1). We used two criteria to account for missing data: (1) we excluded participants with ≥ 90% missing values across *all* dataFields (i.e., all candidate pain variables) and (2) we excluded *specific* dataFields with ≥ 75% missing data across *all* participants (see Supplementary Table 2 and Supplementary Fig. 1 for further details). In addition to age, sex, and body mass index (BMI), a total of 154 pain-related phenotypes across 34,337 participants were available for analysis (cf. Supplementary Table 3). Demographic information for this final 34,337 sample is presented in Supplementary Table 10. We systematically normalized the pain-related variables by recoding each pain experience item from 0 to 10, with 0 being the “best” (reflective of health) and 10 being the “worst” (reflective of disease); see Supplementary Tables 3 and 4 for details and examples. Variable normalization was an important step as the UK Biobank codes used different variables to score similar responses. Following this variable normalization step, we imputed missing data by randomly sampling (with replacement) from existing, non-missing data.

### Multivariate pain-brain decomposition

Pattern-learning algorithms, such as partial least squares (PLS) canonical analysis, provide powerful approaches for extracting meaningful patterns from complex, heterogenous variable sets [34]. PLS is a natural choice for a deeply phenotyped dataset such as the UK Biobank for several reasons, including (1) we expected high autocorrelation between the input dimensions, (2) PLS allows for an effective use of data in the context of high-dimensional, phenome-wide data, as analyzed in our present study, and (3) PLS is highly data-efficient by estimating one coefficient corresponding to each input dimension. These 3 reasons also hold for linear discriminant analysis with the difference that linear discriminant analysis (LDA) is targeted at classification settings, whereas PLS is naturally targeted at explaining variation in continuous outcomes. PLS was implemented in Python package sklearn (version 0.21.3) to decompose the collection of > 150 pain-related participant responses (hereafter, pain features) with respect to cortex regional volumes (hereafter, brain features; cf. Supplementary Tables 1–4). PLS is particularly useful when trying to identify patterns in very large and strongly correlated data, such as in clinical populations where patients often report symptom domains or diseases that overlap [35, 36]. In our previous work, PLS was used to study how loneliness manifests itself in the brain’s anatomy [35] and, separately, how clinical risk factors for social isolation predispose people to dementias [36]. In our study, PLS was thus used to identify the most explanatory projections that yielded maximal covariance between sets of brain volumes in the context of participant reports of pain experience, aiming to yield neurobiologically grounded profiles of pain experience.

Intuitively, PLS has the ability to accommodate two variable sets and thus allows the identification of patterns that describe *many-to-many* relations. Although akin to principal components analysis (PCA), PLS maximizes the linear correspondence between *two* sets of variables. That is, a key feature of PLS is that it identifies the correspondence between two sets of variables, here capturing two different levels of observation—brain structure measurements (hereafter, brain features) and pain-related indicators (hereafter, pain features). Bringing multiple data modalities to the same table is a process that has previously been referred to as *multi-modal fusion* (see Yang et al., 2019 for a review [37]) The PLS algorithm, therefore, seeks dominant dimensions that describe shared variation across different sets of measures. In so doing, PLS can re-express a set of correlated variables in a smaller number of hidden factors of variation. These latent sources of variation are not always directly observable in the original measurements, but together explain dominant motifs of how the actual observations are intrinsically organized.

More technically, assuming *X* and *Y* are centered, the relationship between the two original variable sets (*X* and *Y*) and the derived canonical variates *U* and *V* can be understood as the best way to rotate the left variable set and the right variable set from their original measurement spaces to new spaces in a way that maximizes their linear correlation. The fitted parameters of PLS thus describe the rotation of the coordinate systems: the canonical vectors encapsulating how to get from the original measurement coordinate system to the new latent space, the canonical variates encoding the embedding of each data point in that new space. Similar to PCA, PLS re-expresses data in form of high-dimensional linear representations (i.e., the canonical variates). Each resulting canonical variate is computed from the weighted sum of the original variable as indicated by the canonical vector.

**Fig. 2:**
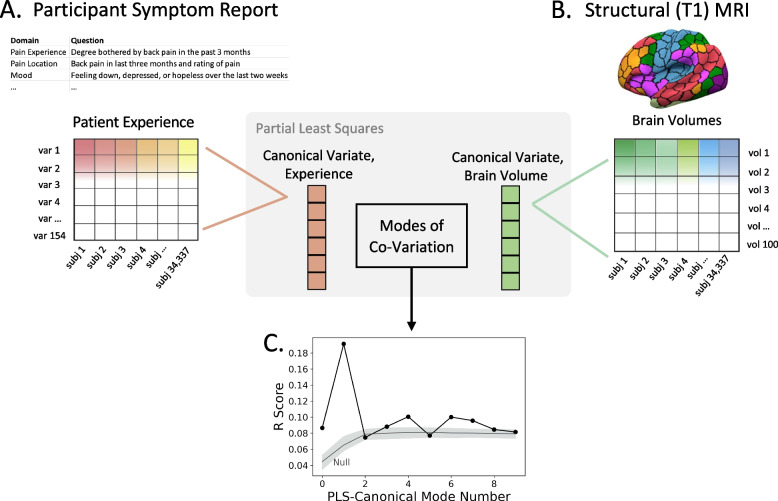
Large-scale analysis of the UK Biobank reveals 4 distinct pain profiles that track distinct domains of pain experience. Our goal was to define profiles of pain experience at a population level. Across 34,337 participants, we gathered 154 pain-related responses (**A**) and 100 anatomically defined brain volumes (**B**), representing a subset of the total UK Biobank (see Supplementary Table 1). We deployed partial least squares canonical correlation as a pattern-learning strategy to trace coherent, biologically driven pain profiles. We identified 4 modes of co-variation that were statistically significant (based on 1000 permutation null distribution shown in grey, panel **C**). For ease of communication, in subsequent figures we refer to modes 1, 4, 6, and 7 as “pain profiles” 1, 2, 3, and 4, respectively. Each pain profile broadly tracked a distinct domain of pain experience (see Fig. 3)

Concretely, to analyze the relationship between the brain features and the pain features, all data were systematically normalized (described above) prior to application of PLS to identify low-rank projections which link variable set $X$ corresponding to the brain features, to variable set $Y$ representing the participant-wise pain features. Two sets of linear combinations of the original variables are obtained as follows:

$$X\in {\mathbb{R}}^{n\times p}$$

$$Y\in {\mathbb{R}}^{n\times q}$$

$$\begin{array}{cc}L_X=XV&L_Y=YU\end{array}$$

$$\begin{array}{cc}l_{x,l}=Xv_l&l_{Yl}=Yu_l\end{array}$$

$$corr\left({l}_{X,l},{l}_{Y,l}\right)\propto {l}_{X,l}^{T}{l}_{Y,l}=max$$

where *n* denotes the number of participants, *p* is the number of brain features (100), *q* is the number of pain features (154), $V$ and $U$ denote the respective contributions of *X* and *Y* to the derived latent “modes” of joint variation between the patterns derived from *X* and patterns derived from *Y*, ${L}_{X}$ and ${L}_{Y}$ denote the individual-level expression of the respective latent “modes,” ${l}_{X,l}$ is the *l*th column of ${L}_{X}$, and ${l}_{Y,l}$ is the *l*th column of ${L}_{Y}$. The goal of our pattern learning approach was to find pairs of latent vectors ${l}_{X,l}$ and ${l}_{Y,l}$ with maximal correlation in the derived latent embedding and quantify the strength of the relationship between the two component sub-groups. Since PLS was purely used as an exploratory analysis, uncertainty in effect sizes were not measured. This step was performed in Python, using SciKit-learn’s PLSCanonical [38]. Statistically significant pain-brain modes of co-variation were defined as *p* < 0.001, based on 1000 permutations (see Fig. 2C), as described below.

To assess the statistical robustness of the PLS-derived modes of pain-brain co-variation, we created an empirical null distribution following a previously published non-parametric permutation procedure [34, 39]. In each of the 1000 permutations, the brain feature matrix remained constant, while the pain feature matrix was randomly shuffled. This approach preserved the statistical structure of the brain features while selectively disrupting the structure of the pain features. The resulting distribution represented the null hypothesis of a random association between brain and pain features across participants. For each permutation iteration, four variables were recorded: the R score for each PLS mode (yielding 10 scores per iteration), pain feature loadings (one per pain feature per iteration), and brain feature loadings (one per brain feature per iteration). These values were used to create null distributions against which the statistical significance of our actual results could be assessed. For example, the Pearson correlation coefficient (rho) was recorded and contributed to the null PLS distribution. *P*-values were derived based on the 1000 rho values from the null PLS model. Multiple comparisons correction was not required, as the study focused exclusively on the leading structural brain variation mode estimated by PLS, which was statistically significant at *p* < 0.05.

**Fig. 3:**
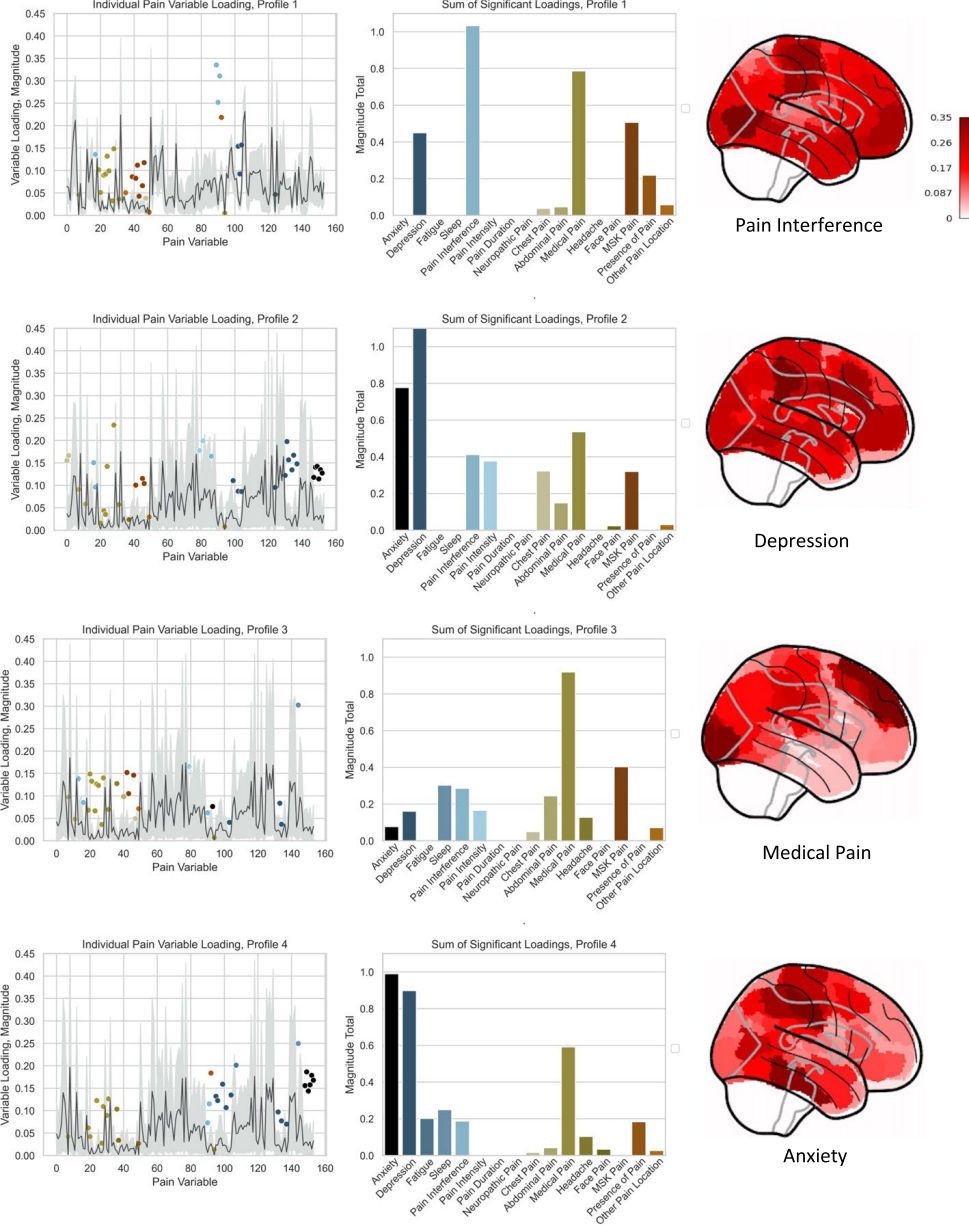
Four pain profiles capture distinct domains of pain experience. For each significant mode (Modes 1, 4, 6, and 7, hereafter referred to here as pain profile 1, 2, 3, and 4, respectively; see Fig. 2), the left panel plots statistically significant loadings in the context of their null distribution. The center panel provides a sum of statistically significant loadings, grouped by domain of pain experience. The right panel shows the relative brain loadings for each mode, which are further presented in “Results” and in Supplementary Fig. 2. We named each pain archetype based on the symptom domain that contributed most to that mode’s loading: profile 1 broadly tracked pain interference symptomatology, profile 2 tracked depression, profile 3 tracked medical pain, and profile 4 tracked anxiety

Given the need to impute missing data (as described above), we conducted a sensitivity analysis to ensure that each pain profile’s characterization was not biased by imputed data. Following the same logic used to define the null PLS distribution, we generated an empirical null distribution for model weights for both pain and brain variables by applying the weights from the PLS permutation analysis (as shown in Fig. 2C). This distribution, shown in Fig. 3 (left column), allowed us to identify pain variables that were significantly loaded, defined as those in the top or bottom 5% of the empirical null distribution based on magnitude (shaded in grey in Fig. 3, left column). We then counted (Fig. 3, middle column) and summed (Fig. 3, right column) the weights of only the significant loadings within each domain, which collectively characterized each profile.

As a further validation of our pain-behavior profiles, we performed a split-half analysis (analogous to our previous UK Biobank studies (Kiesow 2020, Saltoun 2023)). We chose a split-half analysis given that our goal was to leverage the UKB’s highly unique choice (real-world) and extent (quantity) of pain variables to define clinically actionable pain profiles. We are unaware of any even remotely similarly rich pain neuroscience dataset, which makes an external validation challenging. As such, we performed a split-half analysis to further quantify the stability of our pain profiles. We randomly split the 34,336 participants into two halves across 1000 iterations, using one half to repeat our analysis pipeline and another for a second repetition of our analysis pipeline. To determine the stability of the 4-profile solution in each of these iterations, we then computed the correlation coefficient (rho) between the profiles as derived afresh from the first and second half of the cohort.

### Pain profile clinical features

To investigate which clinical features were associated with each pain profile, we examined the potential associations between individual-level expressions of pain-brain modes and participants’ phenotypic, diagnostic, and medication histories. For this, we used an individual-level output from the PLS analysis, referred to as the “PLS Pain Score.” The PLS Pain Score is represented by an (*n* x *q* × 4) weight matrix, where *n* is the number of individuals, *q* is the number of pain features (154), and 4 is the number of significant loadings. Each weight value in this matrix indicates the relationship of an individual’s pain features to each of the four pain modes. For example, a higher loading (or PLS Pain Score) for a participant on Mode 1, which represents Pain Interference, suggests that this participant has a stronger expression of pain features associated with Pain Interference.

None of the clinical features (phenotypic, diagnostic, or medication histories) were included in the original PLS analysis. Each association with individual-level clinical histories therefore represents an independent profile of each pain mode, and thus an independent validation of the meaningfulness of the derived pain profiles.

*Phenome-wide association study (PheWAS)* of participants’ derived pain scores were defined by reference to a wide variety of almost 1000 lifestyle factors, demographic indicators, and laboratory values. Saltoun et al. recently published this tool to characterize brain asymmetries [40]. Feature extraction was carried out using two utilities designed to obtain, clean, and normalize UK Biobank phenotype data according to predefined rules. Initially, a raw collection of ~ 15,000 phenotypes from 11 major categories was available for analysis. We used the FMRIB UK Biobank Normalisation, Parsing And Cleaning Kit (FUNPACK version 2.5.0; https://zenodo.org/record/4762700#.YQrpui2caJ8) to harmonize and clean these phenotypes using predefined, standard arguments available within the FUNPACK workflow. Cleaning steps included excluding any brain-imaging-derived or mental health-related variables (as these were both related to the PLS analysis), and deploying built-in tools to remove “do not know” responses and unasked dependent data. FUNPACK therefore yielded ~ 3300 phenotypes, which was then input into PHEnome Scan ANalysis Tool (PHESANT), a tool designed specifically for curating UK Biobank phenotypes [22]. The PHESANT toolkit combined phenotypes across visits, normalized, cleaned, and categorized the data as belonging to one of four datatypes: categorical ordered, categorical unordered, binary, and numerical. All categorical unordered columns were converted into binary columns to encode a single response. For example, the employment status phenotype was originally encoded as a set of values representing different conditions (e.g., retired, employed, on disability). The output of categorical one-hot encoding on unordered phenotypes was then combined with all measures classified by PHESANT as binary, numerical, or categorical ordered. By default, FUNPACK assessed pairwise correlation between phenotypes and discarded all but one phenotype of a set of highly correlated (> 0.99 Pearson’s correlation rho) phenotypes. For example, left and right leg fat percentages were highly correlated (Pearson’s rho 0.992). Hence, only right leg fat percentage was included in the final set of phenotypes. Also by default, both FUNPACK and PHESANT exclude variables with fewer than 500 participants; we kept this default. There were 11 categories available for analysis, but because some of the mental health phenotypes may overlap with pain-related responses, we removed all 220 phenotypes belonging to the “mental health” category. Overall, 757 phenotypes from 10 major categories were available for PheWAS.

Each PheWAS examined a battery of associations with subject-wise pain scores with the 757 extracted phenotypes. By computing Pearson’s correlation for each phenotype and pain score pair, we were able to extract both the association strength and accompanying statistical significance of the given phenotype-pain profile pairing. For each profile, two standard tests were used to adjust for the multitude of associations being assessed. First, we used Bonferroni’s correction for multiple comparisons, adjusting for the number of tested phenotypes (0.05/757 = 6.6e-5). Second, we further evaluated the significance our correlation strength using the false discovery rate (FDR), another popular method of multiple comparison correction which is less stringent than Bonferroni’s correction. The false discovery rate [41] was set as 5% and computed for each asymmetry pattern in accordance with standard practice [22, 42]. For visualization purposes, phenotypes in Manhattan plots were colored and grouped according to the category membership defined by FUNPACK.

*Diagnosis-wide Association Study (DiWAS)* of participants’ derived pain scores were defined by reference to a wide variety of almost 1,500 disease phecodes extracted from participants’ electronic health records. Feature extraction for both ICD-9 and ICD-10 diagnoses was carried out using the FMRIB UK Biobank Normalisation, Parsing And Cleaning Kit (FUNPACK version 2.5.0; https://zenodo.org/record/4762700#.YQrpui2caJ8) to extract ICD codes. Once extracted, ICD-9 and ICD-10 codes were grouped into 1477 hierarchical phenotypes which combine related ICD-9 and ICD-10 codes into a single “phecode,” using previously established definitions [43]. In addition to combining related ICD codes, we use built-in exclusion criteria to reduce case contamination of control groups [44]. In our UKB subset of people with both pain variables and brain imaging (*n* = 34,337), 22 diseases were not represented in our subset. We excluded these 22 ICD codes from the final analysis. The final set of 1425 diagnostic phecodes was later compared to discovered pain profile expression to probe for relations between patient-reported pain profiles and clinical diagnoses.

More concretely, we used FUNPACK on the UK Biobank sample to extract phenotype information related to ICD-9 diagnosis codes and ICD-10 diagnosis codes. The FUNPACK utility contained built-in rules tailored to the UK Biobank which resulted in the extraction of a total of 542 ICD-9 codes and 4105 ICD-10 codes. ICD-10 codes contain more granular information than ICD-9 codes, and separate ICD-9 and −10 codes may reflect common etiologies [45]. To consolidate related medical diagnoses across and within ICD coding paradigms, we used the Phecode Map 1.2 with ICD-10 codes made available from https://phewascatalog.org/phecodes_icd10 [43]. The phecode system additionally established clinically guided exclusion criteria relevant to creating a health comparison cohort associated with each phecode. As a specific example, the exclusion criteria associated with “Type 2 diabetes” (phecodes 250.1) includes individuals with related diseases, including Type 1 diabetes and secondary diabetes mellitus, as well as patients with symptoms commonly associated with Type 2 diabetes, such as abnormal glucose [43]. Individuals with related diseases, but not the disease of study, are therefore excluded from the control group based on the built-in exclusion criteria, which may otherwise decrease the statistical power for finding genotype–phenotype associations [44]. The final set of 1425 clinical phecodes spanned 17 disease classes as defined by Wu and colleagues [43], ranging from congenital anomalies and neoplasms, to mental disorders and infectious diseases.

We charted robust cross-links between the subject-wise expression of a given pain profile and the 1425 extracted phecodes, with appropriate correction for multiple comparisons. For each extracted phecode, the Pearson correlation between the given phecode and the inter-individual variation in pain archetype revealed both the association strength and accompanying statistical significance of the given phenotype-pain archetype association. Individuals fulfilling the exclusion criteria defined by Wu et al. (2019) were removed from the control group, separately for each phecode. For each uncovered pain profile, two standard tests were used to adjust for the multitude of associations being assessed. First, we used Bonferroni’s correction for multiple comparisons, adjusting for the number of tested phenotypes (0.05/1425 = 3.5e − 5). Second, we further evaluated the significance of our correlation strength using the false discovery rate (FDR), another popular method of multiple comparison correction which is less stringent than Bonferroni’s correction. The false discovery rate [41] was set as 5% [22, 40] and computed for each pain archetype in accordance with standard practice [42]. For visualization purposes, phecodes in Manhattan plots were colored and grouped according to the category membership defined by the phecode map developed by Wu and colleagues (2019).

*Medication-wide Association Study (MedWAS)* of participants’ derived pain scores were defined by reference to the Anatomical Therapeutic Chemical (ATC) classification published by the World Health Organization (www.who.int/tools/atc-ddd-toolkit/atc-classification). The ATC classification groups active substances according to the organ or system on which they act and to their therapeutic, pharmacological, and chemical properties. Drugs are classified in groups at five different levels, based on target organ (level 1), therapeutic subgroup (2), pharmacological subgroup (3), chemical subgroup (4), and chemical substance (5). We chose to use level 3 in our analyses as we were interested in drug category. The UKB includes 6745 different types of drug or supplement entries across 374,891 participants (listed under Data-Field 20,003). Using in-house code, each of 6745 medications was searched for its corresponding ATC codes at each ATC level. This search was performed solely on the medication name and not on the medication dose (see Supplementary Methods, Supplementary Table 7). Because medication dose was not collected by the UKB team (see 12.4, https://biobank.ndph.ox.ac.uk/showcase/refer.cgi?id=100235), all medications were collapsed into medication name independent of dose and formulation. Individual medications were subsequently collapsed into ATC codes thereby providing another step which summarized medications on the individual level. Medication frequency (i.e., how many times per day a medication was taken), treatment duration (how many days a medication was taken), or whether a medication was taken at the time of interview (i.e., that day) was not available for analysis.

Because some medications fall into more than one ATC category depending on which dose or route of administration is used (e.g., finasteride, see whocc.ni/atc/structure_and_principles/), we selected the first ATC category in alpha-numeric order. Medications listed in the UKB that did not have corresponding ATC codes were excluded from analysis (e.g., many dietary supplements, free text entries). Medications which were combinations of two drugs (e.g., budesonide and formoterol are found together as an inhalant for COPD) were excluded from analysis. Given that our analysis revealed an association between opioids and pain interference, we noted that the ATC classification of codeine was unclear in some instances. Codeine is an opioid that (in combination with paracetamol or aspirin or ibuprophen) can be used as an analgesic; however, because it is a weak opioid, it is most often used as a cough suppressant (R05D). To not bias our results, we insured that codeine was *not* included under the larger category of N02A Opioids. We did note that insulin was listed in the UKB as “insulin product” (coded as 1,140,883,066); because this did not specify which type of insulin, we coded this ATC Level 3 Category “A10A Insulins and Analogues.” Overall, the UKB medication data represents 14 ATC Level 1 Domains (all of them), 73 Level 2 categories, and 137 Level 3 categories. At the individual participant level, medication profiles were coded as follows: the total number of medications a participant was prescribed within each ATC category was represented as a whole number. For example, a participant who reported taking escitalopram, bupropion, and oxycodone would correspond with an ATC Level 3 profile would be a 137-place vector with a “N06A Antidepressant” value of 2 and an “N02A Opioid” value of 1.

Each MedWAS examined a battery of associations with subject-wise pain scores with the 137 ATC Level 3 codes. By computing Pearson’s correlation for each ATC code and pain score pair, we were able to extract both the association strength and accompanying statistical significance of the given medication-pain profile pairing. For each profile, two standard tests were used to adjust for the multitude of associations being assessed. First, we used Bonferroni’s correction for multiple comparisons, adjusting for the number of tested phenotypes (0.05/137 = 3.64e − 4). Second, we further evaluated the significance our correlation strength using the false discovery rate (FDR), another popular method of multiple comparison correction which is less stringent than Bonferroni’s correction. The false discovery rate [41] was set as 5% and computed for each asymmetry pattern in accordance with standard practice [22, 42].

*Regarding opioid and antidepressant versus pain interference and mood disorder*,we wanted to better understand the observation that opioids and antidepressants were associated with different profiles of pain interference and mood disorder. Across the 502,507 participants in the UK Biobank, we restricted our analysis to those who had data about medication, pain interference (the Brief Pain Inventory, or BPI), and mood (the Patient Health Questionnaire 9, or PHQ-9). Similar to our original criteria, we excluded participants with either ≥ 90% missing data and further excluded dataFields with ≥ 75% missing data. This resulted in 167,203 participants. We then imputed missing data by randomly sampling without replacement using the same procedure reported above. To facilitate visualization, for each individual, we then used established methods to sum, round, and bin the total score: the BPI into mild (1–4), moderate (5–6), and severe (7–10) categories; the PHQ-9 into minimal (1–4), mild (5–9), moderate (10–14), moderately severe (15–19), and severe (20–27) categories. These data were visualized as a matrix of pie charts.

### Comparison of pain profiles with classic body part framework

We next sought to provide evidence that the four pain profiles add information beyond the classic body part framework. We used LDA to train and test models that predict individuals’ dominant pain profile or painful body part (class definition described below) based on behavioural and lifestyle factors used in the PheWAS. LDA was a natural choice of method as it allows the prediction of multiple classes from a large set of features which, in this case, comprised brain structure and real-world behavioural and lifestyle measures. Below, we describe class definition and performance measurement.

#### Class definition: painful body part

We converted the UKB’s pain location question into body part labels as described by the International Association for the Study of Pain (IASP) Axis 1: Regions. Given that our intent was to associate each participant’s phenotypes with the body part of worst pain, whenever an entry in Data-Field 120,037 (“area most bothered by pain in last 3 months”) was present, this was used as default. When 120,037 was not present, we mapped UKB’s Data-Field 120,021 (“Pain or discomfort all over the body in the last 3 months”) to IASP’s 900 (“More than three major sites”). We further mapped all participants who had more than a single body part identified in the Data-Field 6159 as IASP’s 900. We did this because, except for the 69,917 participants who responded to question 120,037, the UKB did not identify which body part was *most* painful, but instead simply listed them in numerical order. 157,042 people identified > 1 painful body part. For those with only two body parts identified, we selected as the primary location UKB response 6159–0.1 (or the first response). Mappings of these codes are presented in Supplementary Table 8 [46]. Counts shown in Supplementary Table 8 are based on the UKB’s Data Showcase website, accessed 3/20/2023: https://biobank.ndph.ox.ac.uk/showcase/field.cgi?id=6159.

#### Class definition: dominant pain profile

Individuals were classified according to the pain profile which most strongly reflects their experience of pain. As not all individuals are expected to be in clinically relevant amounts of pain, individuals which do not strongly express any pain profiles were categorized into a separate no pain class. The proportion of individuals in the no pain class was informed by the number of people who experience no pain in the location-based approach.

By construction, the developed pain profiles rely on pain-location information (cf. above). As we were interested in comparing pain experience to pain location, we elected to use modified pain scores which exclude pain location information in the LDA analysis. To do so, we zeroed-out all data fields which recorded pain location before applying the pain profile definitions obtained through the PLS analysis ($U$ in the equations above). The resultant “pain-agnostic” individual-level pain scores maintain the correlation structure between data-fields established from the full dataset, but do not contain information about individual-level pain location.

To facilitate the interpretations of individual level pain scores, we flipped the signs of pain profiles such that the resultant pain-agnostic pain scores are positively correlated with increasing amounts of location-based pain. Through this procedure, we are ensured that increasing individual level scores are associated with increasing bodily pain, though the pain-agnostic pain scores themselves do not contain any body-location pain information. Note that flipping the sign does not change the magnitude of covariance between pain variables, eigenvalues of the pain profiles, or interindividual variation in pain score. Flipping the sign is purely to aid interpretation.

All individuals express all pain profiles to various extents. To assign individuals to particular pain profile which most closely reflects their experience of pain, all individuals were categorized according to their largest pain score. For example, an individual that expresses the pain interference pain profile more strongly than any of the other three pain profiles would belong to the pain interference class through this procedure. Through this approach, everyone belongs to one of four pain profile classes. However, we know that not all individuals are in clinically relevant amounts of pain. We therefore leveraged information from the pain-location based approach, wherein 34.0% of individuals reported no pain in any location, to establish an additional class of individuals with no pain. The 34.0% of individuals with the smallest pain scores across all pain profiles were grouped into a new “no pain” class instead of their respective “strongest” pain profile. These resultant 5 classes therefore reflect the pain/no-pain distribution seen in the location-based approach and categorize individuals according to their experience of pain rather than where it is located.

#### Cross-validation and model performance

To improve the generalizability of the LDA models, we used a fivefold out-of-sample cross validation wherein the available data were divided into 5 equal samples, trained on 4/5, and tested on the left out 1/5. Class distribution was maintained across all splits, such that all classes were represented in the held out test set for all five splits. Given substantial class imbalance observed in dominant pain profile and painful body part membership (see above), data was resampled to balance the classes prior to fitting the LDA, and was tested on held-out data with and without resampling. For each training iteration, 10 separate resampling protocols were performed for a total of 50 iterations across splits and samples. Performance was measured as accuracy (computed as correctly predicted class labels divided by the total number of labels). We further report Area Under the Curve (AUC) using a micro-averaging approach (AUC(micro)). AUC(micro) is a common method for evaluating multi-class classification performance. AUC(micro) calculates AUC by pooling together true positive rate and false positive rates across all classes using class size as weights [47–49].

## Results

### Identifying biologically grounded profiles of pain experience

We sought to establish biologically valid profiles of everyday pain experience across the UK Biobank (*n* = 34,337) population (see Fig. 1A). Although the demographic profile of the UK biobank is an area of active, ongoing research [21], we present demographic information for this subset and for the full 500,000 UK biobank sample in Supplementary Table 10. We supplemented the Pain Experience Questionnaire with other relevant pain, depression, and anxiety inventories from the UK Biobank, for a total of 154 responses per participant. We further integrated 100 brain volumes per participant based on the Schaefer-Yeo atlas. As a pattern-learning strategy, we deployed PLS to identify coherent pain modes of co-variation driven by primary brain biology itself (*p* < 0.001, based on 1000 iterations in an empirical permutation analysis, see Fig. 2C).

Four statistically significant pain profiles emerged in our data-driven investigation, linking real-world reports of pain experience to individual differences in brain structure. For each significant mode, we report (and illustrate in Fig. 2C) the actual R score and the low–high 95% CI of the null distribution for each significant mode: Mode 1 (0.191 (0.058–0.076)), Mode 4 (0.100 (0.074–0.087), Mode 6 (0.100 (0.075–0.086), Mode 7 (0.096 (0.074–0.085). These four pain profiles were confirmed to be stable across 1000 randomly assigned split-half analyses (see Supplementary Fig. 2 for histogram of rho values across analyses).

We inspected which domains of pain experience were most heavily weighted in each pain profile, providing an initial indication for whether each profile captured a unique pattern of pain symptomatology (see Fig. 2, Supplementary Figs. 3 and 4). Indeed, each pain profile captured a different domain of patient-reported pain experience: Pain Interference (Mode 1), Depression (Mode 4), Medical Pain (Mode 6), and Anxiety (Mode 7; see Fig. 2, Supplementary Figs. 3 and 4). Hereafter, we refer to each mode by their primary symptom domain. Sex, age, and BMI showed relevance to all profiles but did not distinguish between any of the four profiles (see MedWAS, below). Pain location and intensity further did not distinguish between any of the four profiles.

To better assess the clinical relevance of the four pain profiles, we performed a series of post hoc analyses that triangulated each pain profile with several complementary assays: (1) medication history, implicating 137 medication ATC Level 3 categories, grouped according to constituent 14 Level 1 ATC Domains, to yield a medication-wide association study (Medication-WAS; or MedWAS); (2) diagnostic history, benchmarking 1425 clinician-assigned ICD codes across 11 disease classes to yield a diagnosis-wide association study (Diagnosis-WAS; or DiWAS); and (3) phenotype history, tapping into 757 phenotypes across 10 phenotypic domains to yield a phenotype-wide association study (Phenotype-WAS). To facilitate comparison of association strengths across pain profiles, correlation coefficients were transformed to *Z*-scores (Ronald Fisher transformation) and conservatively thresholded using the Bonferroni correction. Together, these high-throughput analytical strategies allowed us to progressively deconstruct the pain profiles in terms of population-level covariation in pain experience and brain volume and individual-level associations with medication, diagnosis, and phenotype.

### Pain profile 1 captures pain interference symptomatology and medications, diagnoses, and phenotypes of chronic disease

The most statistically significant pain profile broadly tracked pain interference. Participants described pain interference in terms of functional limitations to mobility (generally), walking, doing “usual activities”, self-care, and mood (see Fig. 3 for summary and Supplementary Table 3B for full details). While there was not a clear, highly weighted painful body part, the first pain profile captured leg and knee pain, osteoarthritis (of one or more joints), migraine, gout, and diabetes. While there were no brain regions specific to any pain profile, the bilateral default mode network, the right control network, the right somatomotor network and bilateral visual systems showed prominent involvements (compared to other brain networks, see Supplementary Fig. 4).

In terms of the individual-level associations with clinical data, medication-WAS (screening 137 indicators) linked participant expressions in this first unique pain profile to systematic associations with medications used to manage pain (opioids), cardiovascular disease (blood pressure modifiers), metabolic disease (glucose, lipid, and cholesterol-lowering agents), gastroesophageal reflux disease, and migraine. In an independent population-level assay, diagnosis-WAS (screening 1425 indicators) confirmed the association with arthropathies (not specific to any body part), cardiovascular disease (hypertension, ischemic heart disease), and metabolic disease (diabetes mellitus, hyperlipidemia, hypercholesterolemia). Phenome-WAS (screening 757 indicators) showed associations with largely modifiable risk factors of chronic disease including physical measures of body habitus (e.g., leg, arm, whole body mass), average household income, disability allowance, employment status, walking pace, substance use (alcohol and smoking), cardiovascular indices (blood pressure, carotid intima-medial thickness), and blood indicators associated with cardiovascular (lipid, cholesterol), metabolic (glycated hemoglobin (A1C), glucose), nephrologic (creatinine), hepatologic (aspartate aminotransferase), rheumatologic (urate (a compound related to gout)), and endocrine disorders (testosterone, glycated hemoglobin (A1c, a measure used in diabetes management)). Overall, none of the pain profiles associated a clinician-assigned diagnoses (or medications) with a specific body part, and instead represented medications, diagnoses, and phenotypes associated with modifiable, chronic diseases. Further results can be referenced in Supplementary Fig. 5 (MedWAS), Supplementary Fig. 6 (DiWAS), and Supplementary Fig. 7 (PheWAS).

### Pain profile 2 captures depression symptomatology

The second, complimentary pain profile tracked predominantly depression symptomatology. Participants described depression in terms of difficulty concentrating, lack of interest or pleasure in doing things, psychomotor slowing (“changes in speed/amount of moving or speaking”), feelings of inadequacy, and impact on normal roles (see Fig. 3 for summary and Supplementary Table 3B for full details). In addition to these driving effects, pain intensity and interference played important roles, specifically in relation to the impact pain had on mood, enjoyment of life, sleep, general activity, work, and relationships. In addition to the bilateral default mode network, the right control network, the right somatomotor network and bilateral visual systems associated with the first pain profile, the second pain profile showed relevant implications for the bilateral somatomotor network and the left salience/ventral attention system (compared to other brain networks, see Supplementary Fig. 4).

Medication-WAS showed associations with drugs used to manage mood (anti-depressants), as well as cardiovascular (blood pressure modifiers), metabolic (anti-gout medications, glucose and lipid-lowering agents), and gastroesophageal reflux disease. Diagnosis-WAS confirmed the association with cardiovascular disease (hypertension, ischemic heart disease) and metabolic disease (diabetes mellitus, hyperlipidemia, hypercholesterolemia). Phenome-WAS showed similar associations with factors associated with chronic disease including physical measures of body habitus (e.g., leg, arm, whole body mass), substance use (alcohol and smoking), and blood pressure. Further results can be referenced in Supplementary Fig. 5 (MedWAS), Supplementary Fig. 6 (DiWAS), and Supplementary Fig. 7 (PheWAS).

### Pain profile 3 captures medical pain conditions

The third unique pain profile in turn tracked primarily medical conditions that can cause pain. Leading indicators of pain experienced in the context of medical conditions referenced the presence of osteoarthritis of one or more joints, cancer pain, gout, carpal tunnel, diabetes, and diabetic neuropathy (see Fig. 3 for summary and Supplementary Table 3 for full details). Additional relevant domains include musculoskeletal pain (of leg, knee, and arm), sleep (insomnia), abdominal pain, pain interference, and pain intensity (reporting pain in the present moment). Complimentary to the previous two pain profiles, the third pain profile showed above-average weightings for the bilateral default mode network, the right control network, bilateral visual networks and bilateral somatomotor networks (see Supplementary Fig. 4).

Medication-WAS showed associations with drugs used to manage pain (non-opioid analgesics and antipyretics, including gabapentinoids, non-steroidal anti-inflammatory drugs, and paracetamol/acetaminophen), mood (anti-depressants), metabolic disease (anti-gout medications, glucose and lipid-lowering agents), and female-related hormone changes (estrogens, calcium, bone anti-resorptive drugs). Diagnosis-WAS showed strongest associations with sex-related cancers (female, breast) and metabolic disease (diabetes mellitus, peripheral nerve disorder) as well as associations with menopausal and digestive disorders. Phenome-WAS showed associations with socioeconomic status (rent/owning, employment status), lifestyle (weekly mobile phone usage, sleep quality/duration, time spent driving), and modifiable risk factors of chronic disease including physical measures of body habitus (e.g., leg, arm, whole body mass), substance use (alcohol and smoking), and blood pressure. Similar to the third profile, blood assay measures indicative of metabolic (e.g., cholesterol, urate, creatinine, testosterone, hematocrit/hemoglobin) or endocrine (testosterone, vitamin D, sex-hormone binding globulin) disorders were also associated with the third pain profile. Further results can be referenced in Supplementary Fig. 5 (MedWAS), Supplementary Fig. 6 (DiWAS), and Supplementary Fig. 7 (PheWAS).

### Pain profile 4 captures anxiety

The fourth unique pain profile tracked, primarily, anxiety, which was detected in conjunction with aspects of depression symptomatology. Participants described anxiety in terms of an inability to stop worrying, restlessness, trouble relaxing, and feelings of foreboding. In contrast to depression symptoms described in the second pain profile, here, depression was described in terms of thoughts of death and poor appetite. In addition to these driving effects, symptoms of fatigue, sleep, and medical pain (osteoarthritis, post-herpetic neuralgia, diabetes, gout) were implicated (see Fig. 3 for summary and Supplementary Table 3 for full details). Overall, there was a notable absence of pain-related variables as compared to the other pain profiles, suggesting this profile related primarily mental health concerns. Similar to the previous three pain profiles, the fourth pain profile showed importance for the bilateral default mode network, the right control and right somatomotor networks. Of note, the fourth pain profile was unique in showing prominent implication of the bilateral dorsal attention networks, which was observed in addition to the bilateral default mode and right control networks which were identified in the other pain profiles (see Supplementary Fig. 4).

Medication-WAS showed associations with drugs used to manage mood (anti-depressants), rheumatologic conditions, metabolic disease (anti-gout medications, glucose and lipid-lowering agents), and cardiovascular disease. Diagnosis-WAS showed strongest association with metabolic disease (diabetes mellitus, gout) as well as associations with rheumatologic and sex-related disorders (menopause, vaginal prolapse). Phenome-WAS showed associations with socioeconomic status (rent/owning, employment status, parent living status, and health), lifestyle (number of sexual partners, pattern balding, exercise, sleep schedule), and modifiable risk factors of chronic disease including physical measures of body habitus (e.g., leg, arm, whole body mass), substance use (alcohol and smoking), and blood pressure. Similar to the third pain profile, blood assay results indicative of metabolic (e.g., cholesterol, urate, creatinine, testosterone, hematocrit/hemoglobin) or endocrine (testosterone, vitamin D, sex-hormone binding globulin) disorders were also associated with the fourth pain profile. Further results can be referenced in Supplementary Fig. 5 (MedWAS), Supplementary Fig. 6 (DiWAS), and Supplementary Fig. 7 (PheWAS).

### Pain profile summary

Each of the four pain profiles captures a different aspect of the pain experience across the population. No profile was specific to any body part (e.g., no profile is specific to “spine pain” or “headache”), whether in reference to the patient’s experience (in the PLS analysis) or to the clinician’s diagnosis (in post hoc, DiWAS analysis). There were clear trends across the profiles, with the medications, diagnoses, and modifiable risk factors for chronic metabolic and cardiovascular disease being interrelated. Across the four pain profiles, we observed notable differences in medications: while the first pain profile capturing pain interference was associated with opioid prescribing, the other three profiles showed a degree of association with antidepressant prescribing.

### Opioid and antidepressant versus pain interference and mood disorder

**Fig. 4:**
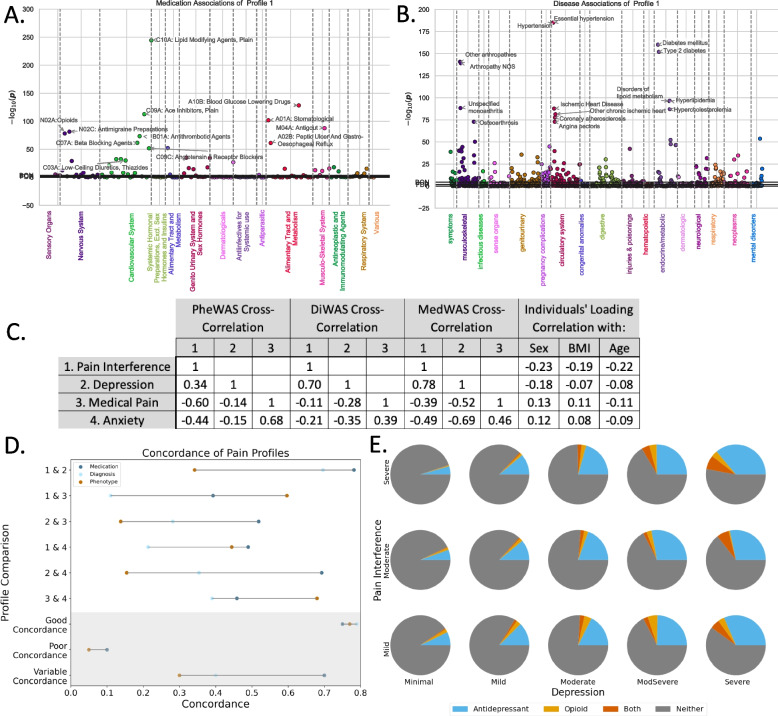
Pain profiles capture distinct aspects of population-level covariation. **A** Diagnosis-wide association studies (Di-WAS, Manhattan plot) relate patterns of pain projections across the population to 1425 clinician-assigned ICD codes across 11 disease classes. **B** Medication-wide association study (Med-WAS, Manhattan Plot) relate pain projections across the population to 137 medication ATC Level 3 categories across 14 Level 1 ATC Domains. In both Med-WAS and Di-WAS, Pearson’s correlation coefficients (between the pain projection and diagnosis or medication of interest) are shown in units on logarithmic scale of the associated *P* value. Horizontal lines indicate the significance thresholds at false discovery rate, Bonferroni correction for phenotypes, and Bonferroni correction for phenotypes and asymmetry patterns together labelled FDR, BON, and BON85 (overlapping in each case). For reference, the first pain profile (that broadly tracked pain interference, see Fig. 3) showed unique, strong associations with medications and diagnoses related to painful conditions (opioids and arthropathies). Associations with cardiovascular and metabolic disease were common to all profiles (see Supplementary Figs. 3–5 and Supplementary Table 9). Similarity matrix (**C**) and a concordance plot (**C**) show that while each of the four pain profiles broadly tracked distinct symptom domains, the first and second profiles (tracking pain interference and depression) and the third and fourth profiles (tracking medical pain and anxiety) were more correlated across medication, diagnosis, and phenotype-wide association studies. All correlation coefficients shown in **C** were statistically significant at *p* < 0.01 level. Overall, **D** concordance plots across different modes indicated that medication and diagnosis are more similar than phenotype. To facilitate visualization, examples of good, poor, and variable concordance are shown at the base of the figure. **E** Prescribing behavior across 167,000 participants shows clear trend that patients report taking an antidepressant, opioid, and both (antidepressant and opioid) in relation to depression symptomatology (measured with PHQ-9, severity of depression increases left to right along the *x*-axis), but not with pain interference (measured with Brief Pain inventory, severity of pain interference increases bottom to top along the *y*-axis)

We performed an additional post hoc analysis to better understand the observation that opioids and antidepressants were associated with different profiles of pain interference and mood disorder. Across a different, non-overlapping subset of the UKB database (*n* = 167,203 participants, see “Methods” for details), we plotted how indices of pain interference (the Brief Pain Inventory, or BPI [50]) and mood (the Patient Health Questionnaire 9, or PHQ-9 [51, 52]) related to three types of medication: opioid, antidepressant, or both (opioid and antidepressant). As shown in Fig. 4E, there is a trend between opioid, antidepressant, and both (opioid and antidepressant) reports with depression symptomatology, but not with pain interference. We therefore observed that worsening mood, but not worsening pain interference, is associated with opioid prescribing in a population of 167,203 participants.

### Concordance across pain profiles

As a high-level summary of the similarity and dissimilarity between the four pain profiles, we correlated the medication, diagnosis, and phenotype WASes of each pain profile (see Fig. 4C). We observed that profiles tracking pain interference and depression were more similar across medication (rho = 0.78), diagnostic (0.70), and phenotypic (0.34) histories; meanwhile, profiles for medical pain and anxiety were more similar across medication (rho = 0.46), diagnostic (0.39), and phenotypic (0.68) histories. Across all pain profiles, a trend was observed for medication histories to be more similar than either diagnosis or phenotype (Fig. 4D), possibly because the same medications can be used for multiple clinical diagnoses and purposes. Taken together, therefore, we note that while the four pain profiles tracked with distinct symptom domains, we describe certain similarities in medication, diagnosis, and phenotype.

### Comparison of pain profiles with classic body part framework

We next sought to ask whether the four derived pain profiles provide information that goes beyond the classic body part framework in today’s clinical practice of pain. We turned to linear discriminant analysis (LDA) as a classification algorithm that can detect and predict principles in data that separates participants into defined groups on the basis of their individual behavioral and phenotypic data. In our case, we sought to compare the relevance of behavioural and lifestyle information in determining group membership, where groups were defined either according to participants’ dominant pain profile, or separately, according to participants’ reported painful body. Therefore, we trained two different classification models, a pain location classifier and a pain profile classifier. The pain location classifier predicted individuals’ painful body part with 16.39% accuracy (± 0.08%; micro-averaged AUC: 0.501 ± 0.007; random chance across 8 classes = 12.5%); meanwhile, our pain profile classifiers trained on the same behavioral and phenotypic data predicted individuals’ dominant pain profile with 26.11% accuracy (± 0.09%; micro-averaged AUC: 0.5903 ± 0.0008; random chance across 5 classes = 20%). Results can be referenced in Supplementary Fig. 8. The pain location classifier’s performance was primarily driven by the model’s ability to distinguish pain of any location from no pain (AUC for no pain location 0.5652 ± 0.0010). The pain location classifier performed poorly when attempting to separate groups based on the specific painful body part (AUC for abdominal pain 0.5110 ± 0.0032; AUC for arm pain 0.5172 ± 0.0024; AUC for back pain 0.4721 ± 0.0067; AUC for head pain 0.5346 ± 0.0022; AUC for leg pain 0.5164 ± 0.0016; AUC for lower back pain 0.4999 ± 0.0021; AUC for cervical region pain 0.5039 ± 0.0014). On the other hand, our pain profile classifier performed better across all groups (whether primarily pain interference (AUC 0.6086 ± 0.0021), depression (AUC 0.5509 ± 0.0011), medical pain (AUC 0.6078 ± 0.0037), or anxiety (AUC 0.5445 ± 0.0013)) and, further, when comparing dominant pain profile to participants with no pain (AUC 0.5457 ± 0.0010). Overall, the improved performance of our pain profile classifier compared to the pain location classifier suggests that our pain profiles offer valuable, additional information and meaningful effects in accounting for real-world behavioral and phenotypic data.

## Discussion

To understand how pain conditions might be best organized, we applied a carefully designed pattern-learning approach to the largest publicly available set of real-world pain experience data. We show that across 34,337 UK Biobank participants, patient-reported experiences of pain fall neatly into four biologically derived profiles. We performed a series of post hoc analyses and show each functional profile was driven by a distinct symptom domain—pain interference, depression, medical pain, and anxiety—each representing a different facet of functional impairment. We further validated that each pain profile reflects real-world, clinically relevant differences in patient function by probing associations of each profile across 137 medication categories, 1425 clinician-assigned ICD codes, and 757 expert-curated phenotypes. We show the relationship of our pain profiles to chronic diseases across a wide range of medical specialties. As a final step, we provide evidence that our four pain profiles add underappreciated cues beyond the classic body-part framework, suggesting that our pain profiles might add value to conversations about healthcare delivery re-design.

Our four pain profiles offer an organizing framework for evaluating and treating pain disorders that is rooted in the functional assessment. Functional status—a summary of a patient’s physical, emotional, and social wellbeing—is a key index of disease risk, treatment outcome, quality of life, and overall healthcare usage across a wide range of clinical settings and patient populations. While the functional assessment is a widely accepted down-stream treatment outcome measure, our results suggest that our pain profiles could instead be deployed upstream, to sort patients into clinical service lines that focus on functional impairment specific to each profile. Here, we propose a function-based operating system in contrast to the classic body-part framework (see Fig. 1 for illustration). The body-part operating system sorts patients into clinical service lines with expertise in a given body part, but not necessarily expertise in improving overall patient function [1]. We further note that optimizing patient function (as opposed to simply improving pain in a specific body part) would require managing the on-going cardiovascular and metabolic diseases that were pervasive across the pain profiles but, currently, do not fall neatly into any given body part. Indeed, our results suggest that patients’ functional and pain outcomes could potentially be enhanced by evaluating and directing patients to treatments targeting chronic diseases, rather than exclusively concentrating on an affected body part. We now discuss each profile in the context of everyday clinical decision making.

Pain interference refers the extent to which pain limits or impedes a person’s ability to carry out daily activities, including work, social, and leisure activities [53]. Of the four profiles, pain interference explains the greatest amount of variance in the PLS model and is the most statistically significant (see Fig. 2C). Of the 137 medication categories, opioids were only linked to the pain profile that broadly tracked pain interference; meanwhile, the remaining three profiles were consistently associated with antidepressant medications. Because pain interference can be viewed as the nervous system’s response to any painful sensations (i.e., not of any specific body part), it is well-established that pain interference is a key aspect of pain management and can significantly impact one’s quality of life independent of which body part is painful [54, 55]. Clinically proven treatments for pain interference are further agnostic of a given body part and include pharmacological, non-pharmacological, and interdisciplinary treatments [55, 56]. Pharmacological treatments for pain interference commonly include opioids, nonsteroidal anti-inflammatory drugs (NSAIDs), and antidepressants [56]. Non-pharmacological interventions include cognitive behavioral therapy, pain reprocessing therapy, and physical therapy, which have been shown to improve pain interference outcomes and quality of life [57–59]. Mindfulness, yoga, and acupuncture further have shown effective outcomes for treating pain interference [55]. Interdisciplinary treatments include pain management programs that include physical therapy, pain psychology, and case management. It is worth noting that treatments for pain interference are not specific to any body part, but instead modify the way the nervous system appraises and manages painful sensations.

While depression, anxiety, and pain co-occur frequently, they may represent different facets of the same disease process. We observed that, in profiles 2 and 4, symptoms of depression and anxiety were observed together as predominant symptom domains (see Fig. 3). We do note that profiles tracking depression and anxiety symptomatology appear to capture different facets of patients’ clinical history—evidenced in our concordance analyses (see Fig. 4C and Supplementary Figs. 5–7) wherein profiles tracking pain interference and depression have more similar clinical histories meanwhile anxiety and medical pain have more similar clinical profiles. Studies have shown that pain and depression share common biological pathways, such as abnormalities in serotonin and norepinephrine function [54, 60]. Anxiety is also believed to share underlying mechanisms related to stress response and neurotransmitter dysfunction [61]. Both depression and anxiety can exacerbate pain and interfere with treatment efforts—irrespective of the body part in question. Affective components of the pain experience are well documented, including recent studies that drew relationships with affective pain and obesity in both complex regional pain syndrome and neuropathic pain [62, 63]. Like pain interference, both depression and anxiety can be effectively treated with pharmacological and non-pharmacological approaches including antidepressant medication and cognitive behavioral therapy [64]. It is worth noting that—today—depression and anxiety are not routinely assessed or treated within the body part operating system.

Medical pain was an unexpected pattern of symptom patterns tracked by our third pain profile. The UKB participants report a variety of painful medical conditions (e.g., rheumatoid arthritis, complex regional pain syndrome, cancer pain, fibromyalgia, migraine; see Supplementary Table 3 for complete list Medical Pain Conditions) and a variety of musculoskeletal pain locations (e.g., neck, shoulder hip, knee pain). While there is ample literature supporting the role of pain interference, depression, and anxiety in the pain experience (described above), we were somewhat surprised to observe a functional profile that captured a heterogenous group of chronic medical and musculoskeletal pain conditions that was not better viewed in the context of pain interference, depression, and anxiety symptomatology. We do note that while pain profile 3 (Medical Pain) had a heavy loading of cancer-related pain, as the FDA has pointed out on numerous occasions, cancer-related pain does not differ substantively from non-cancer pain in terms of the pain signal processing (i.e., nociceptive pain). This could explain why cancer-related pain was observed alongside other medical causes of pain (e.g., gout, diabetes) in our third pain profile. It is possible that the “medical pain” profile simply captures the portion of the UKB cohort that suffers from chronically painful conditions, albeit with less functional deficits related to pain interference or mood dysfunction. A reasonable explanation might be found in Johnston et al.’s recent report that, across ~ 380,000 UKB participants, “Multiside Chronic Pain” (the number of sites of chronic pain) was associated with 76 genome-wide significant SNPs, primarily related to the nervous system [65]. While our sample likely overlaps with that used by Johnston et al., our results align with the conclusion that the nervous system remains the common organ that predisposes, processes, and perceives painful stimuli, independent of mood dysfunction or specific medical condition and, critically here, independent of whether a patient reports a specific body part in relation to their pain.

Neurobiologically, across all four pain profiles, the most commonly observed brain networks were the default mode (DMN), control, and attention networks. A recent coordinate-based meta-analysis of 54 structural brain-imaging studies of chronic pain, reported systematic changes in brain regions involved in the DMN, control, and attention networks, arguing that chronic pain conditions alter brain structure in non-random patterns that resemble large-scale connectivity patterns [66]. While this meta-analysis reports greater “susceptibility” of right-side brain regions in chronic pain, we report largely bilateral loadings within each pain profile, perhaps reflecting our larger sample with greater representation of pain and medical conditions [66]. Most consistent across our four pain profiles was the DMN, which is increasingly recognized for its clinical relevance in disorders like pain interference, depression, and anxiety [67, 68]. Indeed, altered DMN activity has been linked to chronic pain conditions such as chronic back pain, complex regional pain syndrome, and osteoarthritis chronic pain conditions [67]. In depression and anxiety, the DMN has previously shown hyperactivity and increased functional connectivity associated with self-referential thoughts, rumination, excessive worry, and fear [69–71]. Various therapies, including pharmacotherapy (including placebo), neurofeedback, mindfulness-based cognitive therapy, and deep brain stimulation, can modify brain function within the DMN [70, 72].

A strength and limitation of our study is that the substantial amount of data (~ 500 k) represents a single healthcare system: the UK healthcare system. It is both possible and likely that patients in other healthcare systems require tailored pain profiles that include information specific to that geographic location. We do note, however, that socioeconomic factors such as income level were included in our analysis and were involved across our pain profiles, suggesting that socioeconomic factors are related to but do not distinguish between these expressions of pain at the population level. A previous study by Lyall et al. [73] reported a “healthy” bias in UKB imaging subsample. That is, the ~ 50,000 participants who scheduled, arrived at, and participated in the UKB MRI data collection procedures were, overall, more healthy across a range of cognitive, mental health, cardiometabolic, inflammatory, and neurological phenotypes than the ~ 450,000 participants who were not part of the subsample [73]. Lyall et al. note that studies based on the imaging sub-sample alone might underestimate the effects of (in our case) pain experience across the larger population. Mindful of this possibility, we have further characterized the 4 pain profiles we defined based on the UKB imaging subsample across the larger UKB population with our MedWAS, DiWAS, and PheWAS and report informative patterns that relate clinical descriptors to each pain profile.

Another limitation of our analysis includes the temporal sequence in which the UKB data were collected. In some participants, there is a temporal separation between the collection of different variables (i.e., the brain imaging and pain questionnaires might not have been collected contemporaneously). Ideally, all data would have been collected in the same setting, at the same time, but this is simply not the reality of how the UKB data were collected. We further note the fact that clinical observations made during an outpatient encounter (such as symptom profiles) and test results (such as MRI scans) are often days, weeks, and, at times, months apart; it is rare for an outpatient to receive a clinical evaluation and MRI on the same day. In this context, the UKB data are more reflective of real-world clinical data collection than the ideal academic study. Consistent with many other publications deploying the UKB data to understand brain-behavior relationships, we conjointly analyzed the data irrespective of collection timepoint. A corollary to this limitation is the fact that all analyses we report are based on the co-occurrence (or covariation) structure of specific pain phenotypes across the UKB population and should not interpreted to imply causal mechanisms. For example, although diabetes was observed as one medical condition commonly observed as part of Pain Profile 3, this result does not indicate that all patients with diabetes will experience pain (they do not) but simply that diabetes commonly co-occurred in patients with strong loadings in Pain Profile 3.

A comparable strength and limitation of our study is the choice of analytic tools deployed. We chose an agnostic analysis strategy that treated all variables as dependent variables (i.e., by not regressing out age, sex) with the goal of defining pain profiles that reflect real-world pain patients. It is further possible that changes to our specific analytic method (PLS) would have yielded different results. As such, it could be argued that our reported results are mathematical solution to a problem characterized by a large number of variables using a specific method. This is, of course, true for any complex statistical method applied to any sufficiently large number of variables. Notwithstanding, we feel that our pain profiles provide a reasonable starting point for future work on how to best sort patients within the context of discussions about healthcare delivery design.

It is worth noting that due to the lack of suitable external datasets, the study can only perform random split-half analysis within the UK Biobank to verify the stability of pain phenotypes. We also note that correlation-based analyses cannot reveal causal mechanisms. Our association of pain phenotypes with drugs, diagnoses, and phenotypes can reveal some important clues, but we cannot determine their causal relationships. We hope that our work will encourage the collection of future, equally detailed datasets wherein our four pain phenotypes could be replicated in an independent population.

Notwithstanding, these limitations our work motivates multiple lines of future research in clinical operations and public health policy. Overall, our comparison of our pain profiles with the classic body part framework suggest that our pain profiles capture different aspects of a patient’s overall function and health than simply asking “where does it hurt?” The ability to capture a clinically relevant signal has practical implications for clinical operations, particularly within a learning or value-based health system: instead of triaging, diagnosing, treating, and billing patients with pain conditions based on a given body part, that same patient might be administered a standardized functional assessment and, based on which pain profile they fall into, could be triaged to a clinical service line best equipped to address functional deficits specific to that pain profile. Future work could test whether deploying our pain profiles as an operational tool improves patient outcomes compared to the traditional body part system. At minimum, our observation that a “loading” onto one of the four pain profiles can be defined based on a simple questionnaire suggests a need for standardization of the clinical assessment of pain to include measures of pain interference, depression, and anxiety along with the presence of other types of medical pain conditions. Standardized clinical assessments that target our four pain profiles could further detect clinical problems and then sort patients into clinical service lines that, instead of focusing solely on body parts, have expertise in managing conditions such as pain interference, depression, and anxiety. More specifically, we hope that our work can motivate and guide progress towards unraveling the complex interplay of pain experience (summarized at the population-level in our pain profiles), brain function, and genetics.

Pain is a complex and difficult to treat condition. The International Association for the Study of Pain (IASP) defines medical pain as a “subjective sensory and emotional experience associated with actual or potential tissue damage, or described in terms of such damage” [74]. There is a vast and expanding literature that emphasizes the importance of evaluating and treating pain interference, depression, and anxiety—especially in the case of “Chronic Multisite Pain.” By combining all pain conditions, en masse, across a large population, we were able to examine pain as a whole, without limiting our results to any specific body part. The pain profiles that we report provide a way to systematically think about individual patient function in a way that transcends any specific painful body part.

Our report contributes to the active conversation about healthcare delivery re-design [75]. The delivery of medical specialization lies at the core of our report: how do we organize a healthcare delivery system to deploy clinicians with specialist training and knowledge to most effectively address patients’ health problems? In parallel, how does a healthcare delivery system triage and sort patients so they arrive at the clinical service line most equipped to address their health problem? There is obvious value to service lines with a specialist clinical workforce trained to evaluate and treat a specific body part (i.e., DSB, PG, and ZI work in spine pain centers and happily refer patients with pain in their head to the headache clinic). Yet, our results suggest that, when taken as a whole, patient outcomes may benefit from the same clinical specialists organized within the same clinical service lines, albeit with a different operational tool that sorts patients into those service lines. Whereas, currently, the operational tool is simply “Where does it hurt?”, we propose a holistic screening device that can identify a patient’s dominant pain profile rooted in their overall functional status (see Fig. 1). We view our work here as a first step towards organizing a health system around pain profiles that lend themselves naturally to quantification—both at initial evaluation and on longitudinal follow-up. For example, one might imagine that should someone receive treatment for their depression, the weight of their loading on Pain Profile 2 might diminish with repeated, longitudinal assessments. One might further imagine that should that same person later develop abdominal pain related to pancreatic cancer, their behavioral phenotypes would load more strongly onto Pain Profile 3. Therefore, we propose that future work focus on testing whether our pain profiles provide a quantitative surface both to characterize population-level covariation (which we have accomplished in this work and will further validate in the future) and to trace patient-level change in disease state, thereby empowering our health operating system to see beyond any specific body part and instead refocus on patient function.

Chronic disease management cannot be separated from the management of pain disorders. At minimum, our pain profiles suggest that patients should be screened and treated for pain interference, depression, and anxiety in the context of their chronic medical diseases. In particular, we report that while opioids were associated with the pain profile that broadly tracked pain interference score, opioid prescribing was associated with severity of mood and not the severity of pain interference (Fig. 4E). There is the unsettling possibility that, even in a health system which has been largely immune from an opioid crisis, patients are prescribed opioids for worsening mood that, on the surface, appears to be pain-related. It is clear that patients would benefit from a multi-disciplinary evaluation that might parse out pain interference from mood dysfunction; while opioids do have euphoric properties, they are not indicated treatments for mood disorders [76]. Given the literature that patient outcomes benefit from clinical specialization, it is possible that service lines that treat pain interference, depression, and anxiety could yield substantial returns for both the patient and the healthcare system, perhaps greater than service lines that focus solely on a painful body part [1].

## Conclusions

We report that, across the wider UK population, four dominant pain profiles emerge that might offer new organizing principles for the evaluation and treatment of pain conditions in the context of chronic disease management. By demonstrating the relevance of pain conditions to overall chronic disease management, this data-driven strategy is of relevance to all specialties of medicine and to the on-going and future design of healthcare delivery systems.

## Supplementary Information

Additional File 1: Supplementary Fig. 1: Pain Variable Missing Data Summary. Supplementary Fig. 2: Validation Analysis of Pain Profiles. Supplementary Fig. 3: Pain profiles showed distinct patterns of symptom loadings. Supplementary Fig. 4A: 4A. Summary of the brain variables, organized by the 7-network scheme. Supplementary Fig. 4B. Summary of the brain variables, organized by the 17-network scheme. Supplementary Fig. 5. Medication Wide Association Summary (MedWAS) of the four pain profiles. Supplementary Fig. 6. Diagnosis Wide Association Summary (DiWAS) of the four pain profiles. Supplementary Fig. 7. Phenotype Wide Association Summary (MedWAS) of the four pain profiles. Supplementary Fig. 8. Pain profiles contain valuable information not present in body part groups.

Additional File 2: Supplementary Table 1. Overview of number of participants available for each level of analysis. Supplementary Table 2. Excluded Data Fields. Supplementary Table 3A. UKB Pain Variable Description. Supplementary Table 3B. Pain variable loading, sorted by magnitude and organized by pain profile. Supplementary Table 4. Examples of Recoding to standardize data directionality and scale. Supplementary Table 5A. Schaefer-Yeo Anatomical Labels for 7 and 17-Network Schemes. Supplementary Table 5B. Brain Variable loadings for each pain profile. For each profile, we present magnitudes of brain variable loading, sorted in descending value. Supplementary Table 6. Sex, BMI, and Age are significantly associated with all pain profiles in variable ways. Supplementary Table 7. ATC Categories for all medications included in the UKB. Supplementary Table 8. Coding scheme to translate from the UKB to the IASP nomenclature. Supplementary Table 9. Large-scale association studies reveal strong associations of all pain profiles with medications, diagnoses, and phenotypes from multiple disease categories. Supplementary Table 10. Demographic Information for the large, ~ 500,000 participant cohort and for the 34,337 participant cohort, as used to define the pain profiles.

## Data Availability

On publication, code used to perform analyses and create figures shown in this manuscript will be posted on GitHub. Access to the data may be requested at https://www.ukbiobank.ac.uk.

## Abbreviations

ATC: Anatomical Therapeutic Chemical
AUC: Area under the curve
BMI: Body mass index
BON: Bonferroni
BPI: Brief Pain Inventory
DiWAS: Diagnosis-wide association study
FDR: False discovery rate
IASP: International Association for the Study of Pain
IRB: Institutional Review Board
LDA: Linear discriminant analysis
LDA: Linear discriminant analysis
MedWAS: Medication-wide association study
MRI: Magnetic resonance imaging
PCA: Principal components analysis
PheWAS: Phenome-wide association study
PHQ-9: Patient Health Questionnaire
PLS: Partial least squares
UK: United Kingdom
