# Supplementary material for: Pain can’t be carved at the joints: defining function-based pain profiles and their relevance to chronic disease management in healthcare delivery design
Source: BMC Med. 2024 Dec 18;22:594. doi: 10.1186/s12916-024-03807-z (PMC11656997; doi:10.1186/s12916-024-03807-z)

Supplementary Figure 1. Pain Variable Missing Data Summary. To understand how much data was missing and therefore needed to be randomly imputed, we summarized the missing data in two ways: based on how much data were missing across dataFields (ie pain experience variables, shown above) and across participants (i.e. eids, shown below). We note that this was simply a validation of the exclusion criteria we deployed: we excluded dataFields with >75% missing data and participants with >90% missing data (see Online Methods for details).

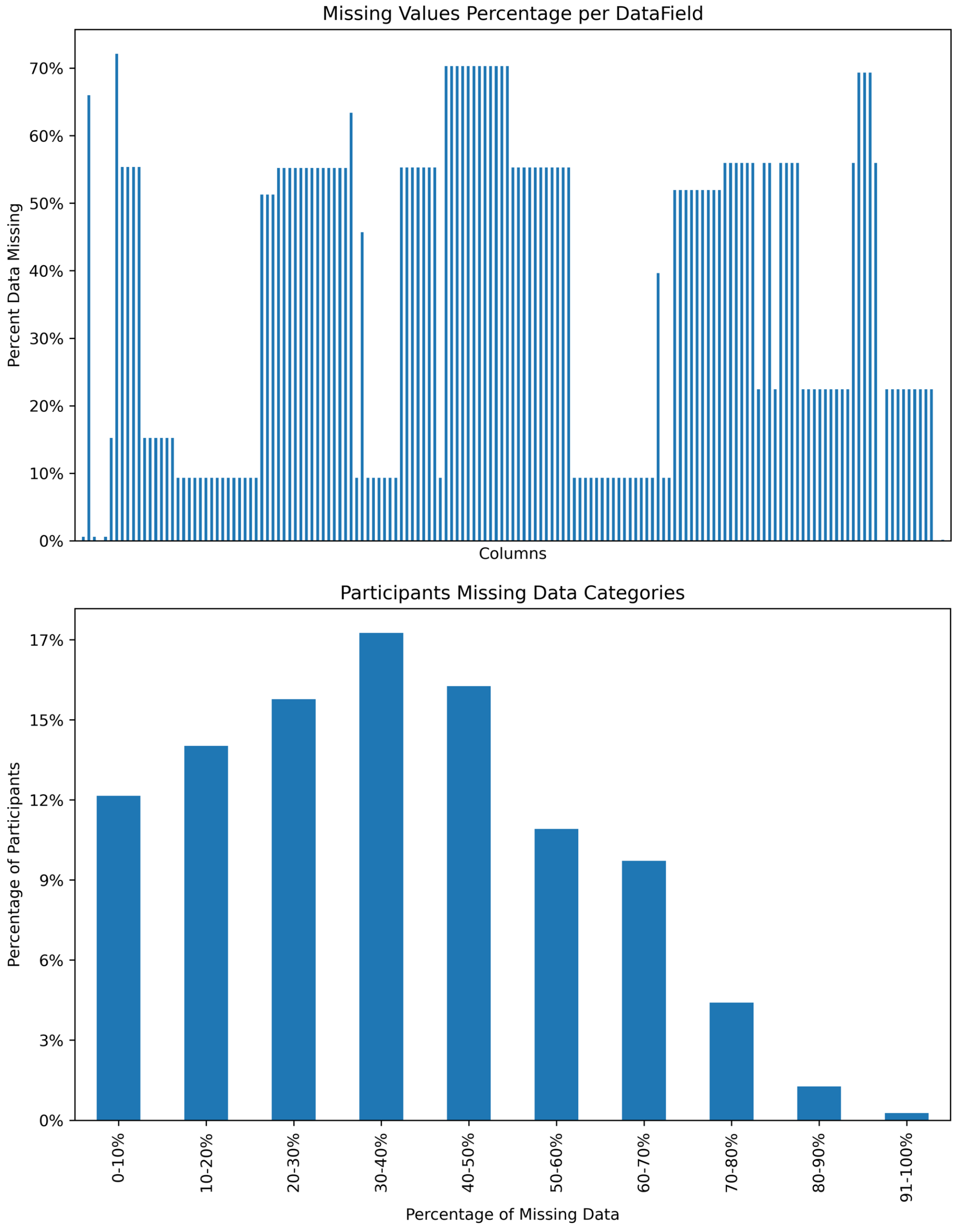

Supplementary Figure 2. Validation Analysis of Pain Profiles. We performed a split-half analysis to further quantify the stability of our pain profiles by randomly splitting the 34,336 participants into two halves 1,000 times, using one half to repeat our analysis pipeline and another for a second repetition of our analysis pipeline. To determine how stable the 4-profile solution in each of these iterations, we then computed the correlation coefficient (rho) between the profiles as derived a fresh from the first and second half of the cohort, considering possible sign flips of the projection vectors (reflection model invariance). Here we present a histogram of the rho values across these 1,000 iterations which demonstrates stability of our models.

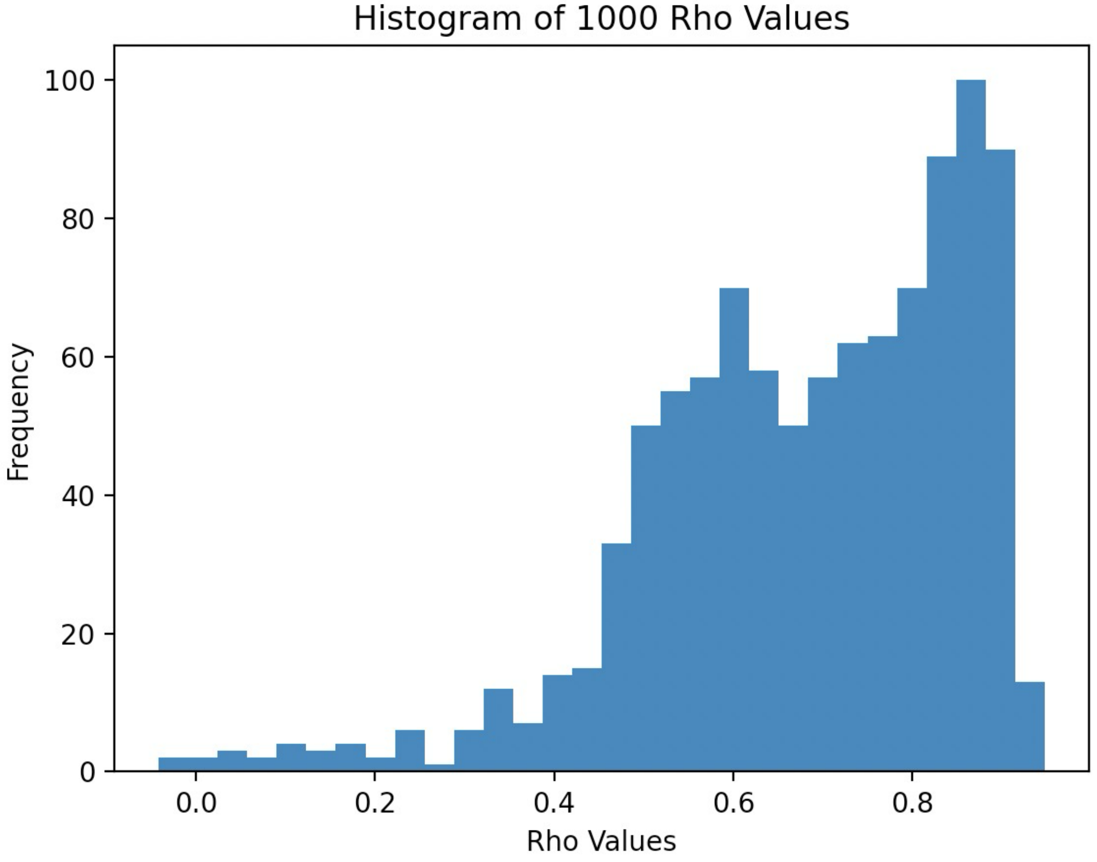

Supplementary Figure 3. Pain profiles showed distinct patterns of symptom loadings. We show relative loadings across 16 different symptom domains for each of the four PLS-C modes. In the main text and figures, these modes are referred to as "pain profiles." As indicated below, modes 1, 4, 6, and 7 are refer to as as "pain profiles" 1, 2, 3, and 4, respectively. These categories were based on the Data-Field labels used by the UK Biobank, see Supplementary Table 3 for complete category breakdown. Each of the four pain profiles captures a unique topography of patient experience, which are summarized in Figure 3, middle panel. Here, we note that pain interference, depression, medical pain, and anxiety broadly track with individual pain profiles. For consistency of reporting, we use the same color dictionary as in Figure 3.

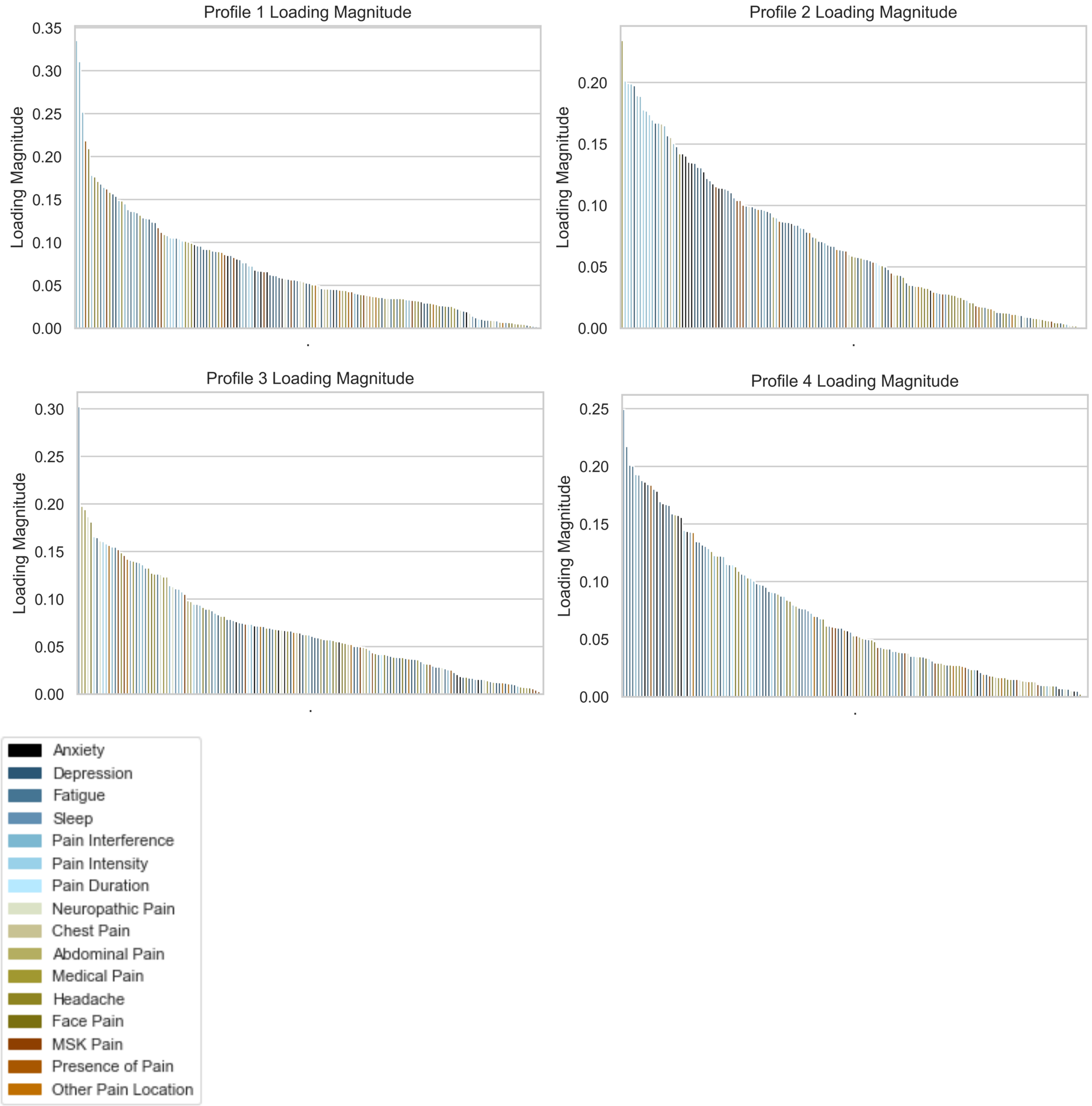

Supplementary Figure 4A. **Summary of the brain variables, organized by the 7-network scheme.** While none of the individual brain variables were statistically significant (based on the null distribution in grey, left column), there were clear trends across modes for above-average weighting of the bilateral default mode network (left more than right), right control network, and bilateral somatomotor networks. The average weighting per profile (across all networks) is indicated with a red horizontal line in the middle column. The right column shows glass brain projections of the magnitude of brain volume loadings specific to each network region. See Supplementary Table 5 for details about the 7-network, 100-node parcellation scheme.

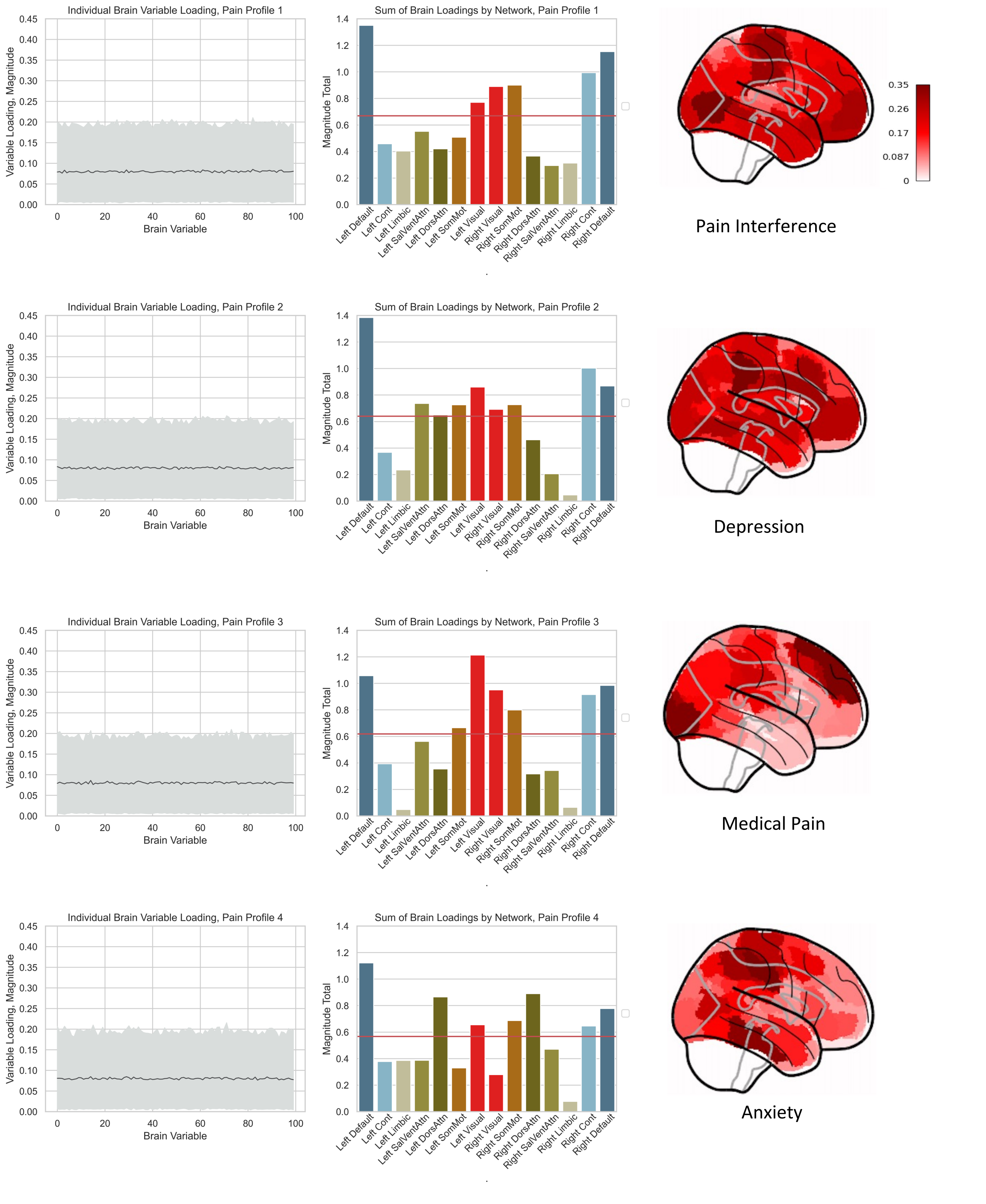

Supplementary Figure 4B. **Summary of the brain variables, organized by the 17-network scheme.** While none of the individual brain variables were statistically significant (based on the null distribution in grey, left column), there were clear trends across modes for above-average weighting of the bilateral default mode network B, dorsal attention networks (A and B), somatomotor network B, and the control network A. The average weighting per profile (across all networks) is indicated with a red horizontal line in the middle column. The right column shows glass brain projections of the magnitude of brain volume loadings specific to each network region. See Supplementary Table 5 for details about the 7-network, 100-node parcellation scheme.

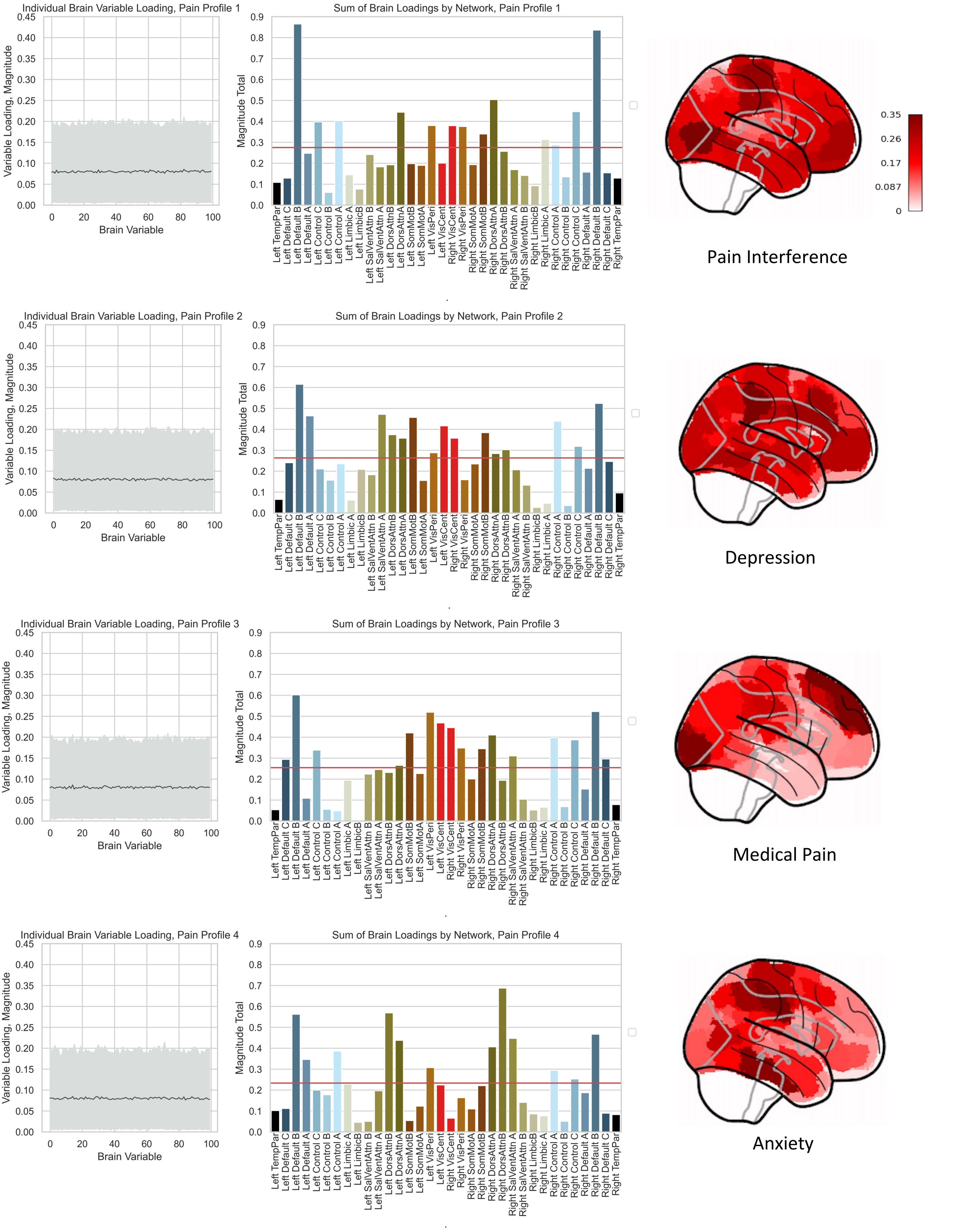

Supplementary Figure 5. Medication Wide Association Summary (MedWAS) of the four pain profiles. For each medication, Pearson's correlation coefficients are plotted in units on logarithmic scale of the associated P value. Horizontal lines indicate the significance thresholds at Bonferroni correction for phecodes (0.05/137), and at FDR correction labelled BON and FDR, respectively. See Supplementary Table 9 for a count of how many medications were statistically significant at the BON and FDR thresholds. Medications treating cardiovascular and metabolic disease were strongly associated with all four pain profiles.

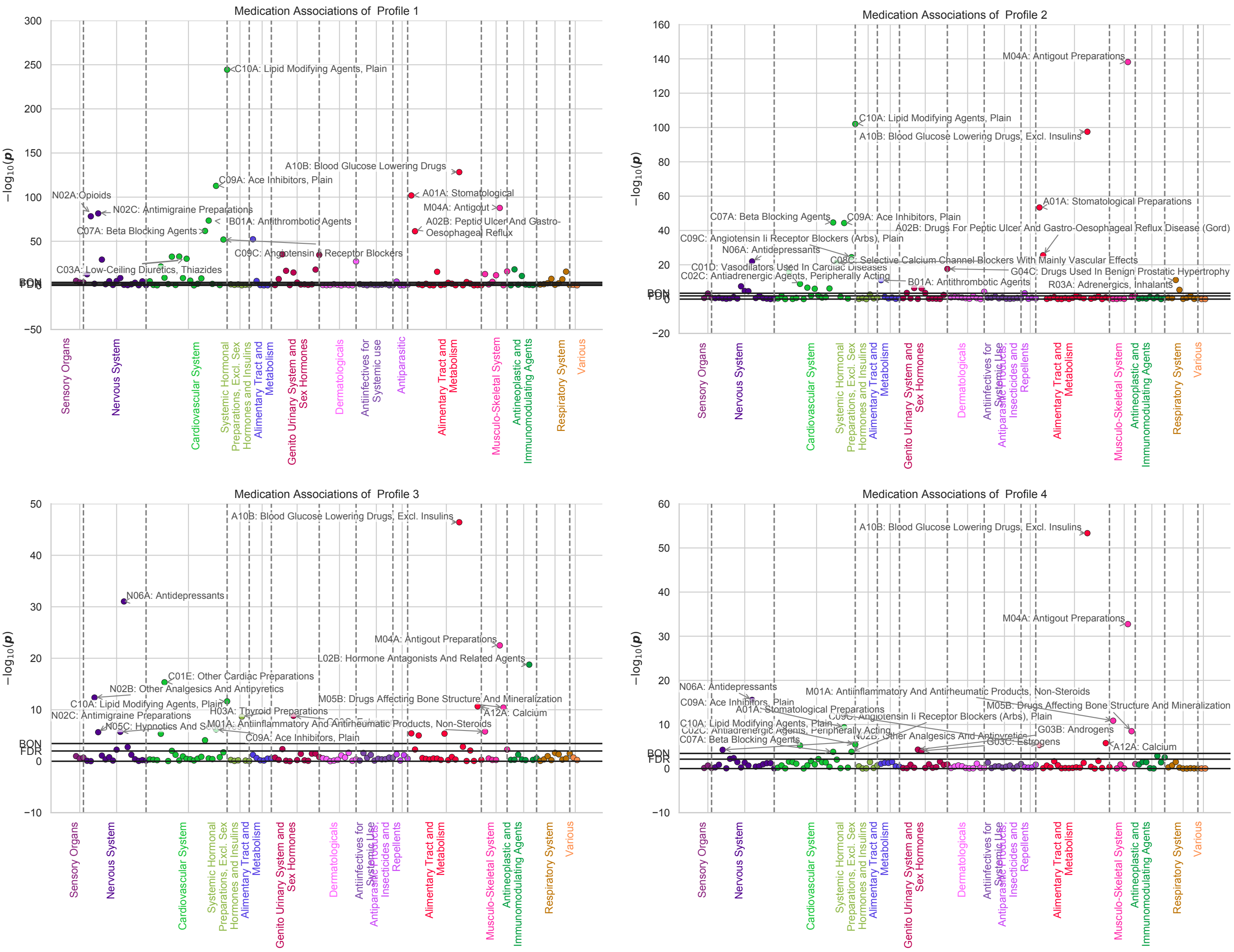

profiles.

Supplementary Figure 6. Diagnosis Wide Association Summary (DiWAS) of the four pain profiles. For each diagnosis (referred to in methods as phecode), Pearson's correlation coefficients are plotted in units on logarithmic scale of the associated P value. Horizontal lines indicate the significance thresholds at Bonferroni correction for phecodes (0.05/1,425), and at FDR correction labelled BON and FDR, respectively. See Supplementary Table 9 for a count of how many diagnoses were statistically significant at the BON and FDR thresholds. Diagnoses related to cardiovascular and metabolic disease were strongly associated with all four pain profiles.

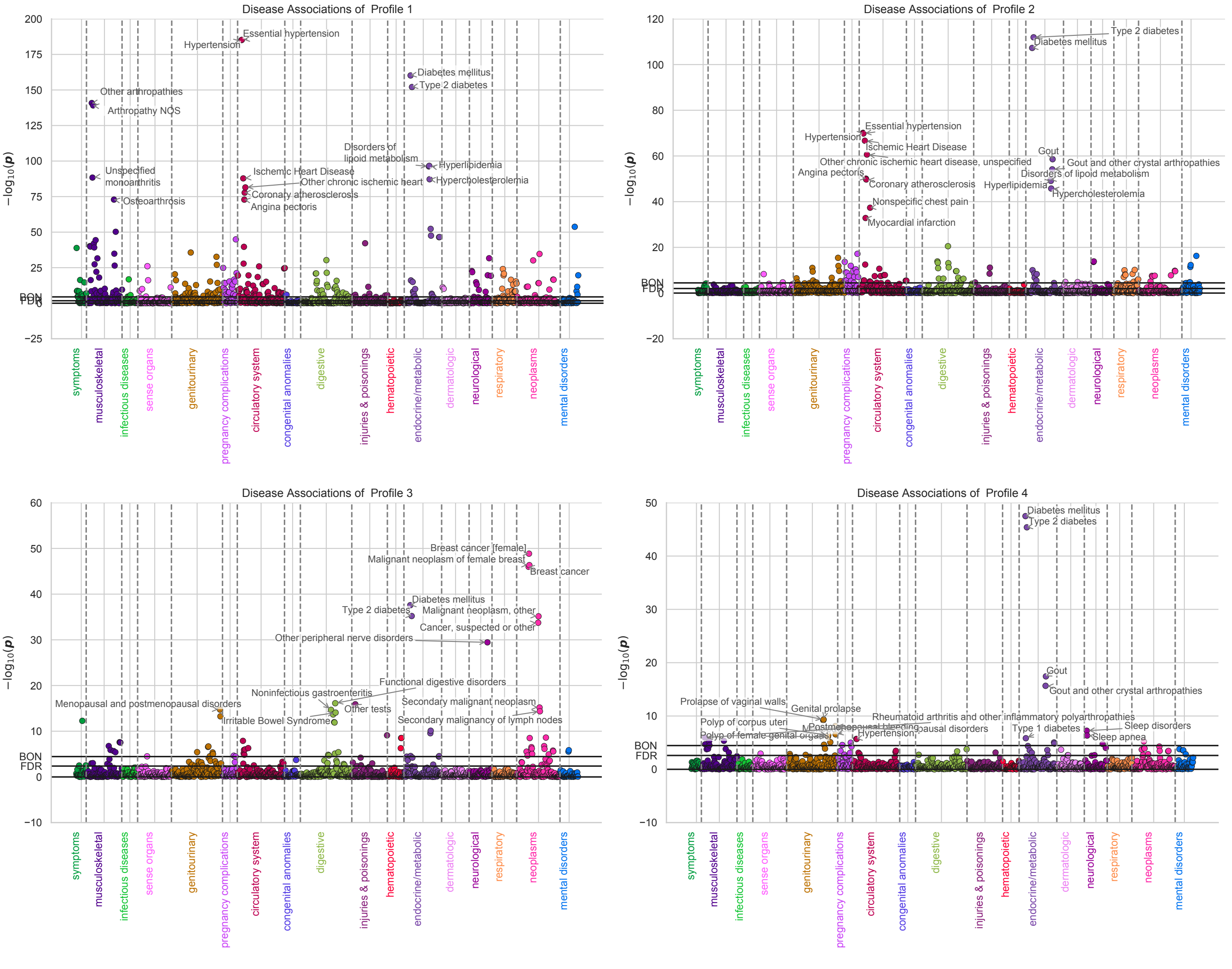

Supplementary Figure 7. Phenotype Wide Association Summary (MedWAS) of the four pain profiles. For each phenotype, Pearson's correlation coefficients are plotted in units on logarithmic scale of the associated P value. Horizontal lines indicate the significance thresholds at Bonferroni correction for phecodes (0.05/757), and at FDR correction labelled BON and FDR, respectively. See Supplementary Table 9 for a count of how many medications were statistically significant at the BON and FDR thresholds. Phenotypes related to cardiovascular and metabolic disease were strongly associated with all four pain profiles.

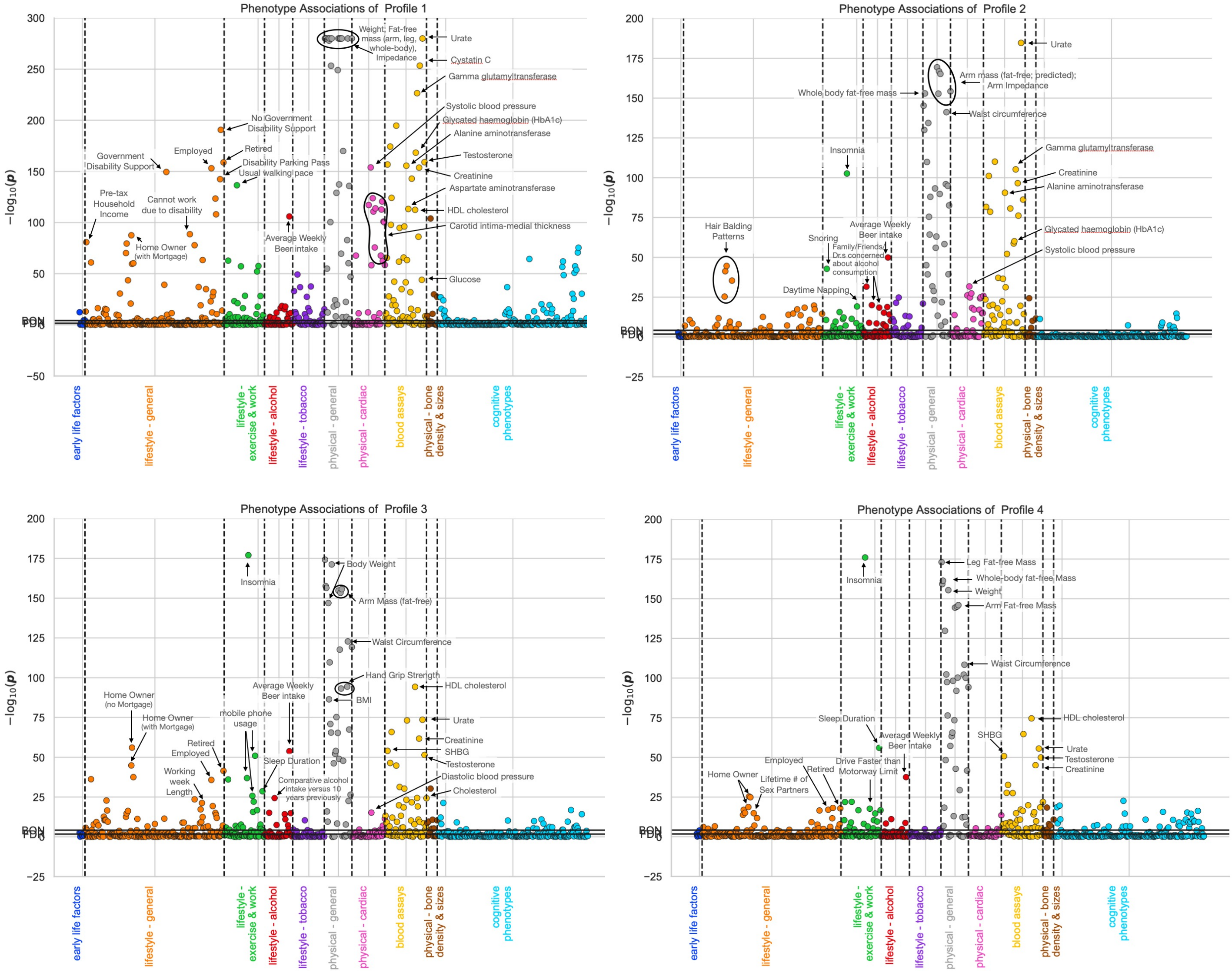

Supplementary Figure 8. Pain profiles contain valuable information not present in body part groups. Linear Discriminant Analysis (LDA) of groups based on painful body part (left) and pain profile (right). Below, we report LDA model performance for specific group classifications when accounted for chance. Painful body part was effective only at identifying participants with no pain but struggled to perform when identifying participants with pain of a specific body part. Groups defined based on painful body part showed an overall accuracy across all 8 groups of 16.39% (chance is 12.5%). Pain profile provided a more useful group category, performing above chance for all groups (noted on diagonal). Pain profile showed an overall accuracy score across 5 groups of 26.11% (random chance is 20%).

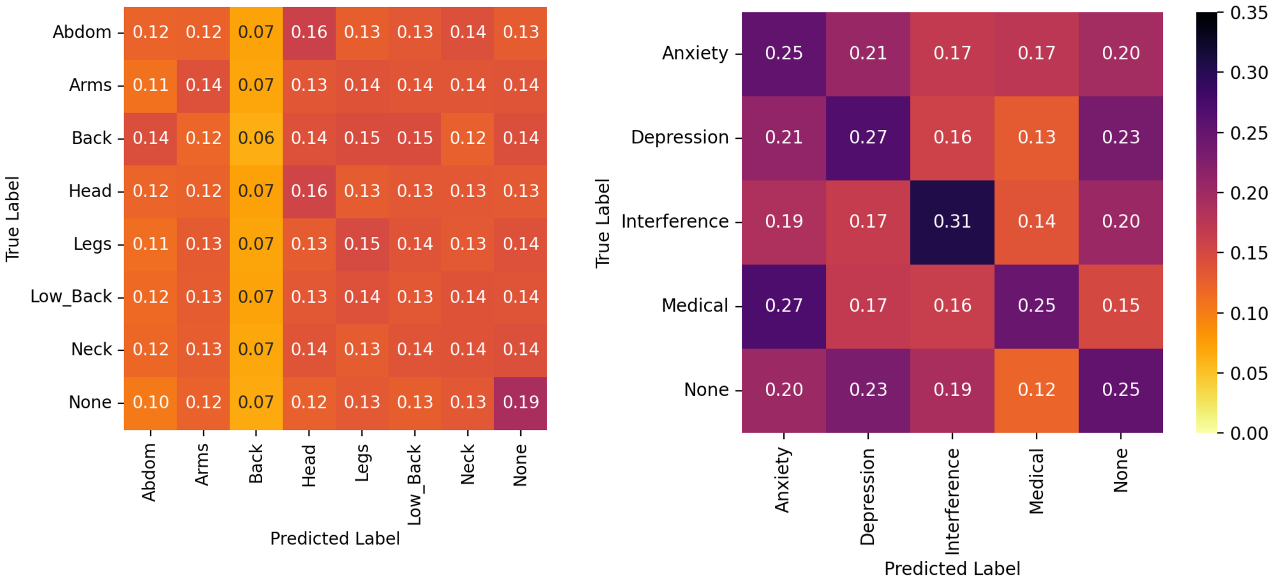

Supplement: Supplementary file 1 — Additional File 1: Supplementary Fig. 1: Pain Variable Missing Data Summary. Supplementary Fig. 2: Validation Analysis of Pain Profiles. Supplementary Fig. 3: Pain profiles showed distinct patterns of symptom loadings. Supplementary Fig. 4A: 4A. Summary of the brain variables, organized by the 7-network scheme. Supplementary Fig. 4B. Summary of the brain variables, organized by the 17-network scheme. Supplementary Fig. 5. Medication Wide Association Summary (MedWAS) of the four pain profiles. Supplementary Fig. 6. Diagnosis Wide Association Summary (DiWAS) of the four pain profiles. Supplementary Fig. 7. Phenotype Wide Association Summary (MedWAS) of the four pain profiles. Supplementary Fig. 8. Pain profiles contain valuable information not present in body part groups. [file 12916_2024_3807_MOESM1_ESM.pdf]
